# Supplementary material for: Elevated levels of FMRP-target MAP1B impair human and mouse neuronal development and mouse social behaviors via autophagy pathway
Source: Nat Commun. 2023 Jun 26;14:3801. doi: 10.1038/s41467-023-39337-0 (PMC10293283; doi:10.1038/s41467-023-39337-0)
Supplement: Supplementary file 1 — Supplementary information [file 41467_2023_39337_MOESM1_ESM.pdf]

Supplementary Information

This document contains the following:

- A complete list of members of BDRL
- Meng Li’s current address
- Supplementary figures 1-19
- Original images of blots for the supplementary figures

Complete members of Birth Defects Research Laboratory (BDRL)

Ian A. Glass'[10], Ian G. Phelps[10], Jennifer C. Dempsey[10], Kevin Lee[1], Lucy Cort[10], Kimberly A. Aldinger[10,11], and Dan Doherty[10]

[10]University of Washington [11]Seattle Children's Research Institute

Meng Li's current address: #Current address: Jiangsu Key Laboratory of Brain Disease and Bioinformation, Research Center for Biochemistry and Molecular Biology, Xuzhou Medical University, Xuzhou 221000, China.

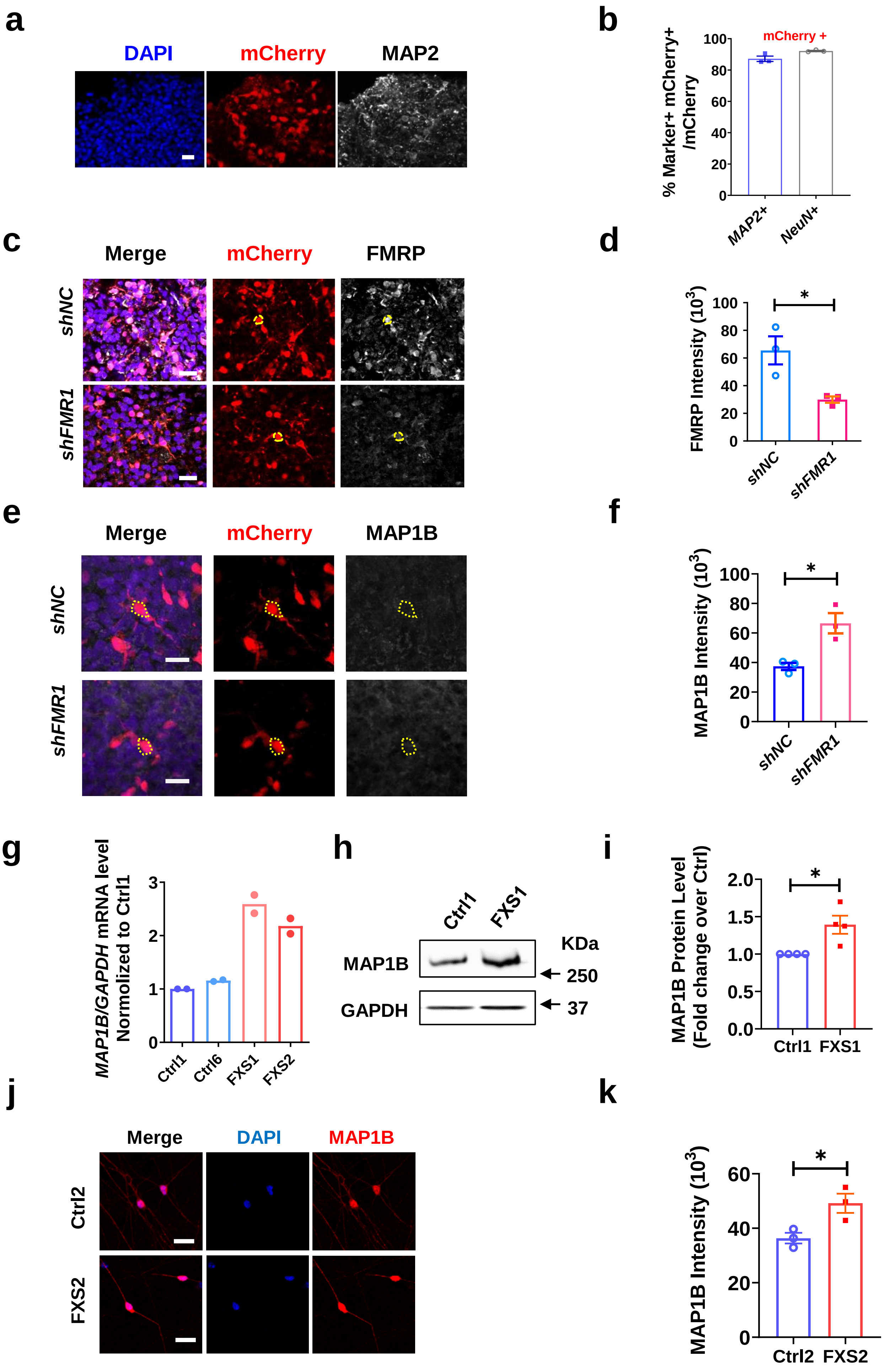
a
DAPI
mCherry
MAP2
b
% Marker+ mCherry+ /mCherry
mCherry +
100
80
60
40
20
0
MAP2+
NeuN+
c
Merge
mCherry
FMRP
shNC
shFMR1
d
FMRP Intensity (10^3)
*
100
80
60
40
20
0
shNC
shFMR1
e
Merge
mCherry
MAP1B
shNC
shFMR1
f
MAP1B Intensity (10^3)
*
100
80
60
40
20
0
shNC
shFMR1
g
MAP1B/GAPDH mRNA level
Normolized to Ctrl1
3
2
1
0
Ctrl1
Ctrl6
FXS1
FXS2
h
Ctrl1
FXS1
KDa
MAP1B
250
GAPDH
37
i
MAP1B Protein Level
(Fold change over Ctrl)
*
2.0
1.5
1.0
0.5
0.0
Ctrl1
FXS1
j
Merge
DAPI
MAP1B
Ctrl2
FXS2
k
MAP1B Intensity (10^3)
*
60
40
20
0
Ctrl2
FXS2

Supplementary Fig. 1 | Elevated MAP1B levels in LV-*shFMR1* infected neurons in rhesus macaque mid-fetal cortical slices and FXS patient iPSCs and iPSC derived neurons. a, Representative immunofluorescence images of human fetal cortical tissue for neuronal marker MAP2. Scale bar, 20 µm. **b,** Quantification of percentage of MAP2-positive and NeuN-positive neurons. N = 3 individual cortices. **c,** Representative confocal images of neurons in the human cortex expressing shRNA-mCherry (red), FMRP (white) in lentivirus-infected ex vivo human cortical slices. Scale bars: 20 µm. **d,** Quantification of FMRP intensity in mCherry+ neurons in macaque cortical slices. Two-tailed, unpaired Student's *t* test, $p = 0.0271$. N = 3 individual cortices. **e,** Representative confocal images of neurons in the rhesus macaque cortex expressing shRNA-mCherry (red), MAP1B (white) in lentivirus-infected ex vivo rhesus macaque cortical slices. Scale bars: 20 µm. **f,** Quantification of MAP1B intensity in mCherry+ neurons in macaque cortical slices. Two-tailed, unpaired Student's *t* test, $p = 0.0157$. N = 3 individual cortices. **g,** qPCR of mRNA levels of *MAP1B* in FXS1 (FX11-7) and FXS2 (FX13-2) iPSCs compared to control Ctrl1 (GM1) iPSCs and Ctrl6 (H13) ESCs. (n = 2 experimental replicates/line). **h,i,** Western blot analysis of MAP1B protein levels in control (Ctrl1) and FXS1 human neurons. GAPDH was used as endogenous loading control and MAP1B protein amounts were normalized to GAPDH and subsequently compared to control cells **(i)**, n = 4 independent differentiation, N = 1 and two-tailed, unpaired Student's *t* test with unequal variances was used, $p = 0.0473$. **j,** Representative confocal images of MAP1B intensity in cultured healthy (Ctrl2, WC6007) and FXS2 neurons. Scale bar, 20 µm. **k,** Quantification of MAP1B intensity in Ctrl2 and FXS2 neurons. Two-tailed, unpaired Student's *t* test, $p = 0.0468$. n = 3 independent differentiation, N = 1. All error bars reflect mean ± s.e.m. Source data are provided as a Source Data file.

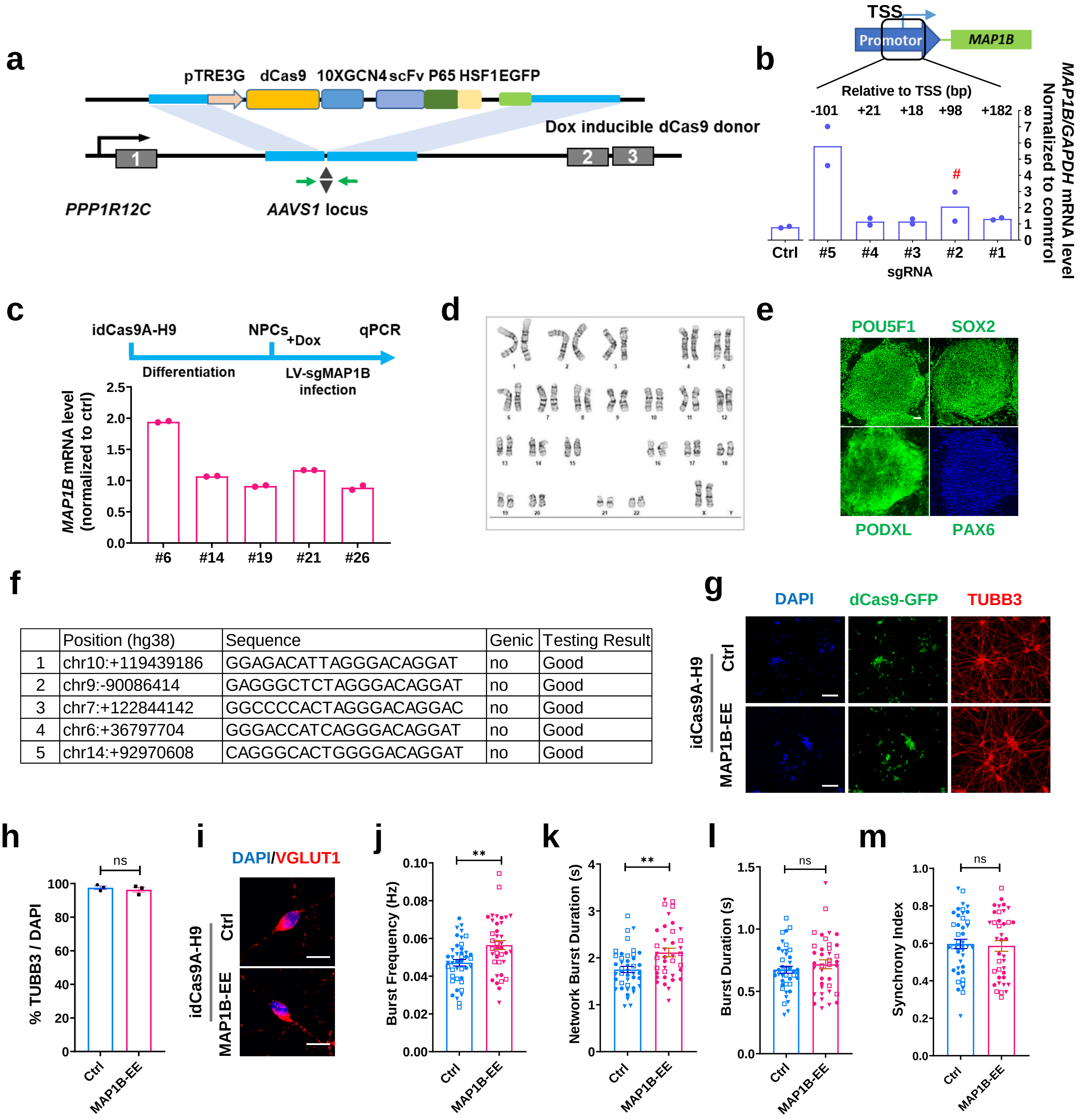

	Position (hg38)	Sequence	Genic	Testing Result
1	chr10:+119439186	GGAGACATTAGGGACAGGAT	no	Good
2	chr9:-90086414	GAGGGCTCTAGGGACAGGAT	no	Good
3	chr7:+122844142	GGCCCCACTAGGGACAGGAC	no	Good
4	chr6:+36797704	GGGACCATCAGGGACAGGAT	no	Good
5	chr14:+92970608	CAGGGCACTGGGGACAGGAT	no	Good

Supplementary Fig. 2 | Creation and characterization of new inducible gene activator human stem cell (idCas9A-H9) lines and MEA analysis of human neurons with MAP1B-EE. a, The genome editing strategy for generating a new inducible targeted gene activator line, idCas9A-H9, in human H9 ESCs. The gene targeting cassette expressing nuclease mutated Cas9 (dCas9) and transcriptional activator complex driven by a tetracycline inducible promoter was inserted into AAVS1 safe harbor site of human genome. **b,** Quantification of mRNA levels of *MAP1B* in the 293T cells transfected with *sgMAP1B* (or *sgCtrl*) with dCas9–VP64 and SAM. Five sgRNAs were designed to target the proximal promoter region (+182 to -101 bp from the TSS) of *MAP1B*. # indicate the sgRNA used in subsequent experiments. We decided not to use #5 because it led to much higher (~7-fold) activation which does not mimic 5q13.2trip. n = 2 experimental replicates, N = 1. **c,** Five isogenic clones (idCas9A-H9 #6, #14, #19, #21, #26) were differentiated into NPCs and infected with LV-*sgMAP1B* #2. The clone #6 exhibited a ~2-fold increase in endogenous *MAP1B* mRNA levels and was used for subsequent experiments. n = 2 experimental replicates, N = 1. **d,** G-banding showing normal karyotypes of idCas9A-H9 #6 ESCs. **e,** Immunofluorescent staining of idCas9A-H9 ESCs for stem cell markers, OCT4 (POU5F1), SOX2, TRA-1-81 (PODXL), and a neuroepithelial marker, PAX6. Scale bar 100 µm. **f,** No off-target mutations detected in the idCas9A-H9 ESCs by Sanger sequencing of top 5 predicted off-target sites. **g,** Representative immunofluorescence images of neurons for neuron marker, TUBB3. Scale bar, 100 µm. **h,** Quantification of percentage of TUBB3-positive neurons. Two-tailed, unpaired Student's *t* test, $p = 0.5328$. n = 3 independent neuronal differentiations, N = 1. **i,** Representative immunofluorescence images of MAP1B-EE and control neurons expressing glutamatergic neuron marker VGLUT1 (SLC17A7). Scale bar, 10 µm. **j-m,** MEA analysis of idCas9A-H9 with MAP1B-EE. Quantifications of burst frequency **(j)**, $p = 0.0023$. Quantifications of network burst duration **(k)**, $p = 0.0016$. Quantifications of burst duration **(l)**, $p = 0.329$. Quantifications of synchrony index **(m)**, $p = 0.7846$. Two-tailed, unpaired Student's *t* test. Ctrl: n = 42 individual wells, MAP1B-EE: n = 38 individual wells from 3 independent neuronal differentiations, N = 1. All error bars reflect mean ± s.e.m. Source data are provided as a Source Data file.

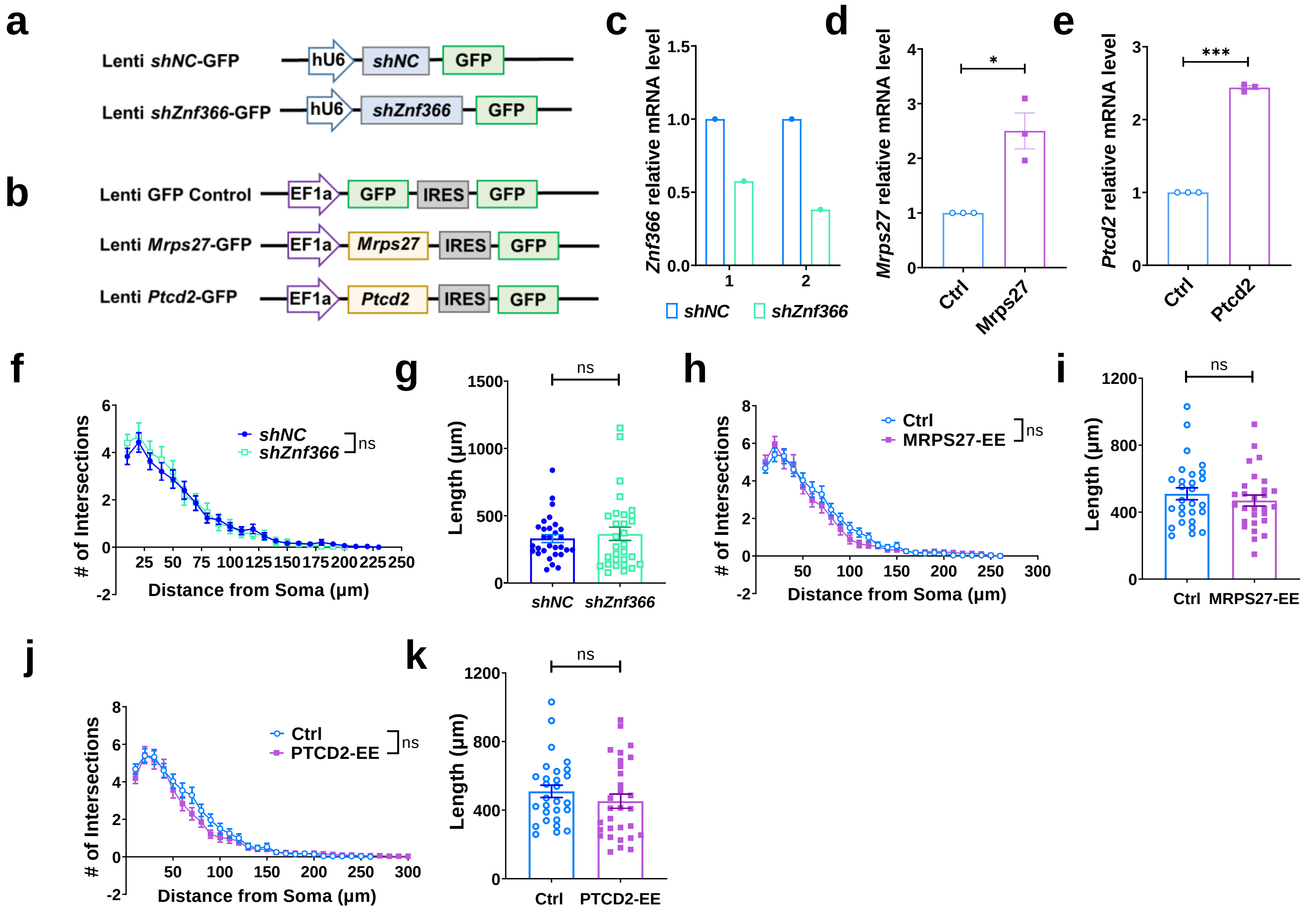

Supplementary Fig. 3 | Assessment of genes affected by 5q13.2trip in neuronal morphological maturation. a,b, A schematic illustration of lentiviral vectors (LV) used for in vitro transduction: LV expressing control *shNC* serves as the control for and LV-*shZnf366*. LV expressing GFP serves as the control for LV expressing MRPS27-GFP or PTCD2-GFP. **c,** Assessing knockdown efficiency of *shZnf366*: qPCR of mRNA levels of *Znf366* in the Neuro2A cells transfected with LV-*shZnf366* or LV-*shNC*. n = 2 technical replicates, N = 1. **d,** Quantification of mRNA levels of *Mrps27* in the Neuro2A cells infected with LV-*Mrps27* compared to cells infection with LV-Ctrl. Two-tailed, unpaired Student's *t* test, n = 3 technical replicates, N = 1, p = 0.0447. **e,** Quantification of mRNA levels of *Ptcd2* in the Neuro2A cells infected with LV-*Ptcd2* compared to cells infection with LV-Ctrl. Two-tailed, unpaired Student's *t* test, n = 3 technical replicates, N = 1, p = 0.0004. **f,g,** Morphological analysis of dendrites of hippocampal neurons with knockdown of *Znf366* (*shZnf366)* compared to controls (*shNC*), assessed by Sholl analysis of dendritic complexity MANOVA, $F_{(1,58)} = 0.062$, p = 0.804, n = 30 cells **(f)**, total dendritic length, p = 0.5649, n = 30 cells **(g)**. **h,i,** Morphological analysis of dendrites of hippocampal neurons with elevated MRPS27 compared to controls (Ctrl), assessed by Sholl analysis MANOVA, $F_{(1,54)} = 0.547$, p = 0.436, n = 28 cells **(h),** total dendritic length, p = 0.4183. Two-tailed, unpaired Student's *t* test. n = 28 cells, **(i)**. **j,k,** Morphological analysis of dendrites of hippocampal neurons with elevated PTCD2 compared to controls, assessed by Sholl analysis. MANOVA, $F_{(1,56)} = 0.892$, p = 0.349. Ctrl: n = 28 cells; PTCD2: n = 30 cells **(j)**, total dendritic length, p = 0.3067, number of dendritic nodes, $*p < 0.05$, n = 30 cells **(k)**. All error bars reflect mean ± s.e.m. Source data are provided as a Source Data file.

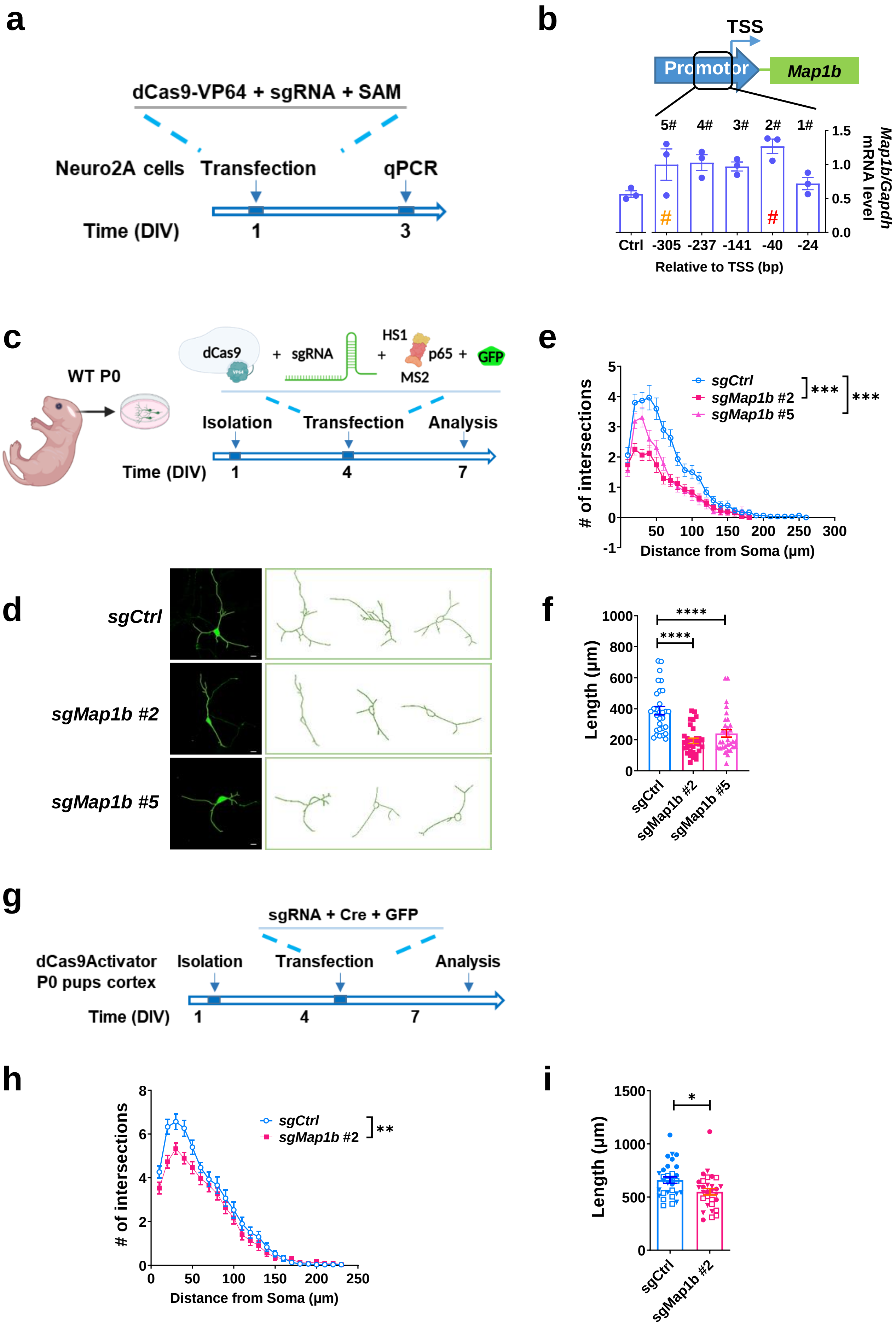

a
dCas9-VP64 + sgRNA + SAM
Neuro2A cells
Transfection
qPCR
Time (DIV)
1
3
b
TSS
Promotor
Map1b
5#
4#
3#
2#
1#
Ctrl
-305
-237
-141
-40
-24
Relative to TSS (bp)
Map1b/Gapdh mRNA level
0.0
0.5
1.0
1.5
c
WT P0
dCas9
VP64
sgRNA
HS1
p65
MS2
GFP
Isolation
Transfection
Analysis
Time (DIV)
1
4
7
d
sgCtrl
sgMap1b #2
sgMap1b #5
e
# of intersections
Distance from Soma (μm)
sgCtrl
sgMap1b #2
sgMap1b #5
***
***
f
Length (μm)
****
****
sgCtrl
sgMap1b #2
sgMap1b #5
g
sgRNA + Cre + GFP
dCas9Activator
P0 pups cortex
Isolation
Transfection
Analysis
Time (DIV)
1
4
7
h
# of intersections
Distance from Soma (μm)
sgCtrl
sgMap1b #2
**
i
Length (μm)
*
sgCtrl
sgMap1b #2

Supplementary Fig. 4 | MAP1B-EE in primary hippocampal and cortical neurons both lead to impaired dendritic development. a, Schematic diagram showing *Map1b* gene activation in Neuro2A cells transfected with *sgMap1b* (or *sgCtrl*) together with dCas9–VP64 and SAM vectors. **b,** Quantification of mRNA levels of *Map1b* in the Neuro2A cells. Five sgRNAs were designed to target the proximal promoter region (–305 to −24 bp from the TSS) of *Map1b*. n = 3 technical replicates, N = 1. Data were analyzed using two-tailed, unpaired Student's *t* test. # indicate the 2 sgRNAs used in subsequent experiments. **c,** Experimental scheme for assessing the dendritic morphology of primary hippocampal neurons. Neurons were transfected with dCas9-based transcription activators of *Map1b*, which consists of three components: dCas9–VP64 fusion protein, synergistic activation mediator (SAM, MS2–p65–HSF1) and sgRNA. DIV, days in vitro. **d,** Representative confocal images (from 3 independent experiment) and Neurolucida software-created traces of GFP+ hippocampal neurons. Scale bar, 10 µm. **e,** Sholl analysis of dendritic complexity of hippocampal neurons with MAP1B-EE (*sgMap1b* #2 or *sgMap1b* #5) or control (*sgCtrl*). MANOVA, *sgCtrl* vs. *sgMap1b* #2: $F_{(1,59)} = 31.151$, $p < 0.001$; *sgCtrl* vs. *sgMap1b* #5: $F_{(1,60)} = 17.038$, $p < 0.001$. *sgCtrl*: n = 30 cells, *sgMap1b* #2: n = 31 cells, *sgMap1b* #5: n = 32 cells. **f,** Quantification of total dendritic length of primary hippocampal neurons. *sgCtrl*: n = 30 cells, *sgMap1b* #2: n = 31 cells, *sgMap1b #5*: n = 32 cells. Data were analyzed using one-way ANOVA with Dunnett post hoc tests, $p < 0.0001$. **g,** Schematic diagram showing isolation and analysis of primary cortical neuron from dCas9-Activator mice. **h,** Sholl analysis of dendritic complexity of mouse cortical neurons with MAP1B-EE (*sgMap1b* #2) and controls (*sgCtrl*). MANOVA, $F_{(1,58)} = 9.385$, $p = 0.003$, n = 30 cells from N = 3 independent isolation. **i,** Quantification of total dendritic length of primary cortical neurons with or without MAP1B-EE. n = 30 cells from 3 independent isolation (N = 3). Data were analyzed using two-tailed, unpaired Student's *t* test, $p = 0.0114$. All error bars reflect mean ± s.e.m. Source data are provided as a Source Data file.

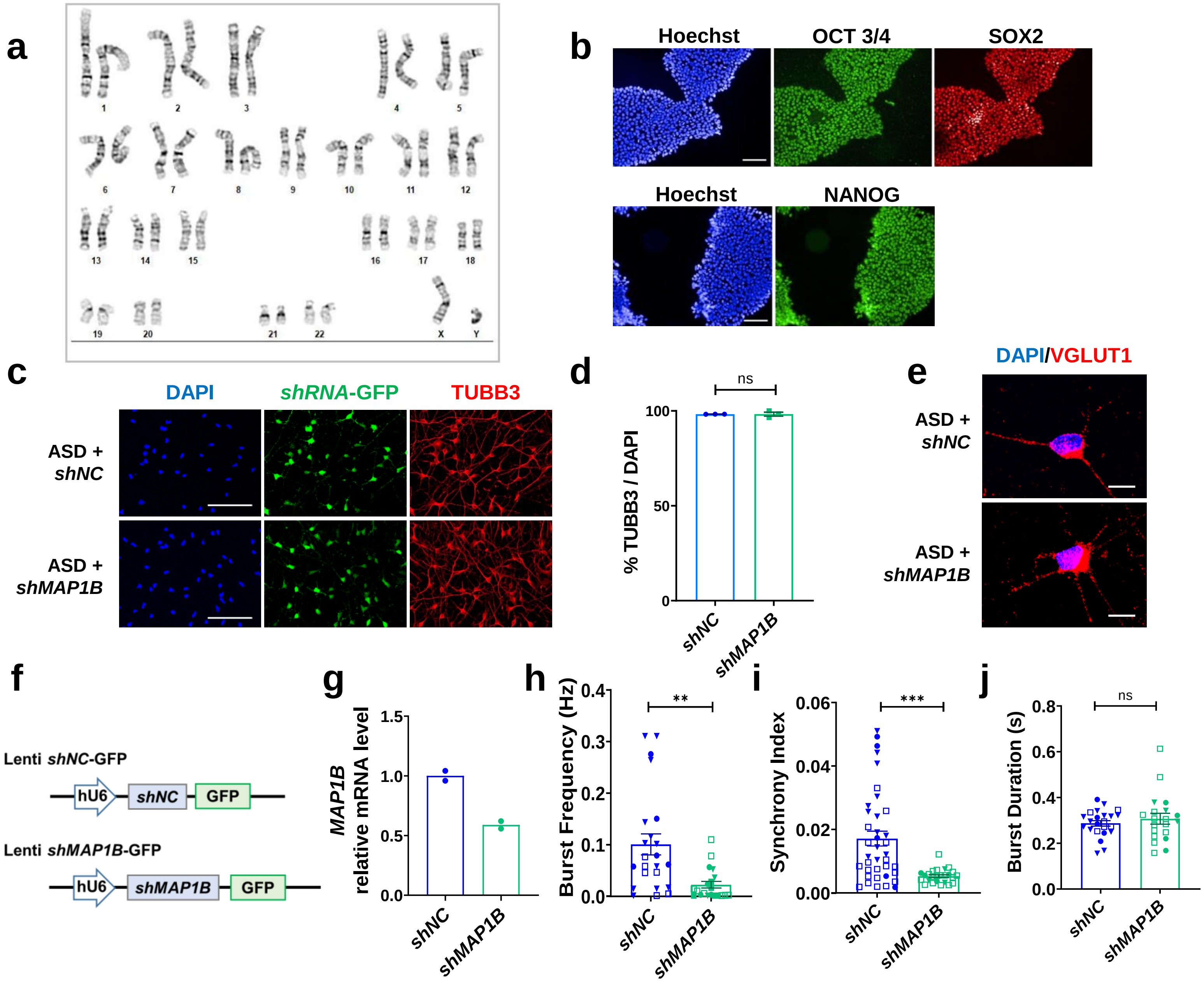

Supplementary Fig. 5 | Characterization of iPSCs derived from the 5q13.2trip ASD patient and evaluation of the rescue effect of *MAP1B* gene knockdown in ASD neurons. a, G-banding showing normal karyotypes of ASD patient iPSCs. **b,** Immunofluorescent staining of ASD patient iPSCs for stem cell markers, OCT3/4, SOX2, NANOG. Scale bar, 100 µm. **c,** Representative immunofluorescence images of neurons for neuron marker, TUBB3. Scale bar, 100 µm. **d,** Quantification of percentage of TUBB3-positive neurons. Two-tailed, unpaired Student's *t* test, p = 0.9539. n = 3 independent neuronal differentiation, N = 1. **e,** Representative immunofluorescence images (from 3 independent neuronal differentiation) of 5q13.2trip patient iPSC differentiated dorsal forebrain excitatory neurons stained with glutamatergic neuron marker VGLUT1 (SLC17A7). Scale bar, 10 µm. **f,** A schematic illustration of LV expressing shRNAs. **g,** qPCR of mRNA levels of *MAP1B* in the 293T cells transfected with *shMAP1B* and *shNC* (n = 2 technical replicates, N = 1). **h-j,** MEA analysis of 5q13.2trip ASD patient neurons with MAP1B knockdown. Quantifications of burst frequency, p = 0.0015 **(h)**. Quantifications of synchrony index, p = 0.0001 **(i)**. Quantifications of burst duration, p = 0.4472 **(j)**. Two-tailed, unpaired Student's *t* test. In (**h**) and (**j**), only the wells with bursting activity were analyzed for burst-related parameters, *shNC*: n = 23 individual wells, *shMAP1B*: n = 20 individual wells from 3 independent neuronal differentiations, N = 1. In (**i**), *shNC*: n = 38 individual wells, *shMAP1B*: n = 25 individual wells from 3 individual neuronal differentiationsm, N = 1. All error bars reflect mean ± s.e.m. Source data are provided as a Source Data file.

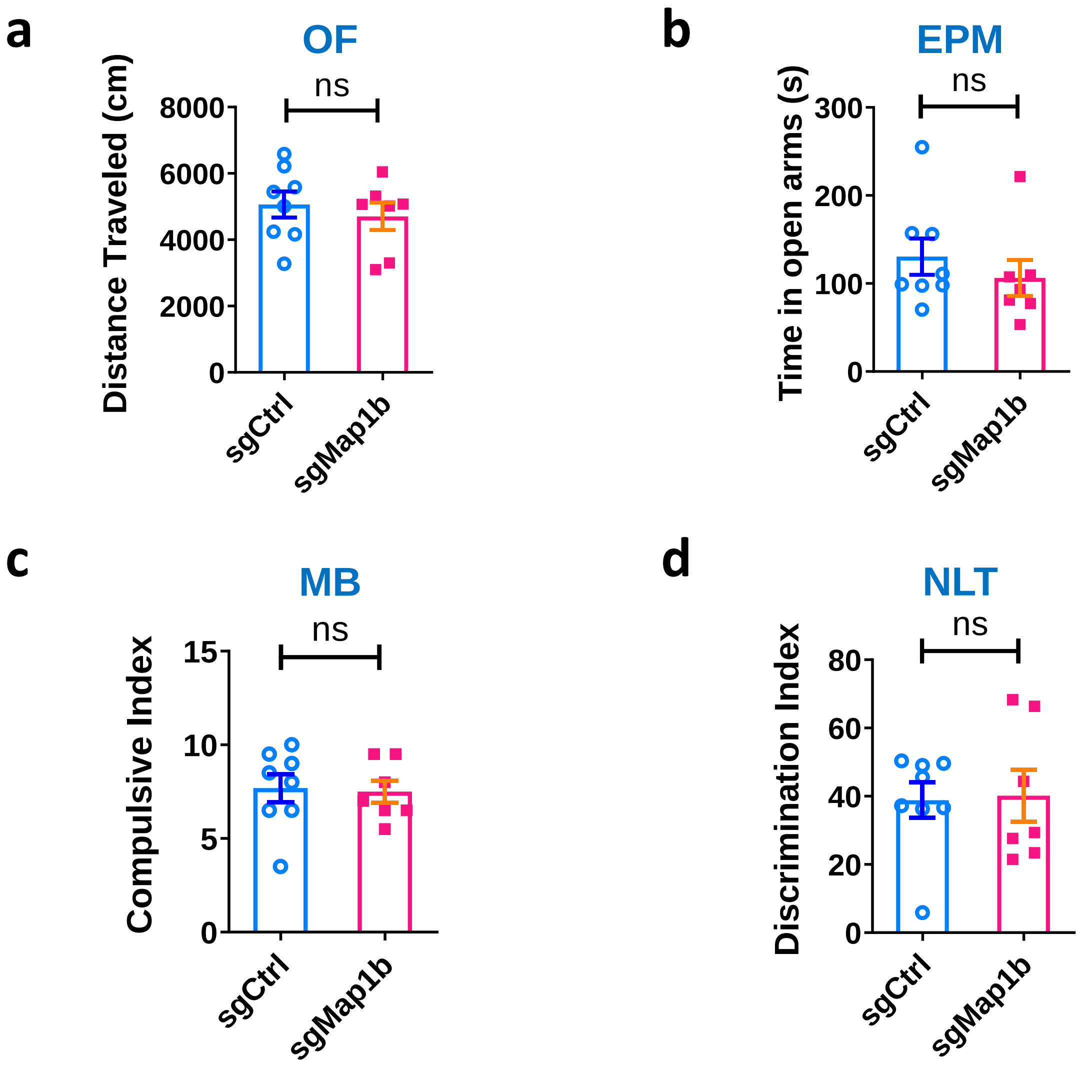

Supplementary Fig. 6 | Targeted activation of MAP1B in excitatory neurons of the prefrontal cortex of mice have no significant impact on overall activities, compulsive behaviors, and spatial learning.

a, Overall activity levels assessed by open field activity (OF) test, $p = 0.5403$. **b,** Anxiety levels assessed by elevated plus maze (EPM) test, $p = 0.4224$. **c,** Compulsive levels assessed by marble burying (MB) test. $p = 0.8502$. **d,** Spatial learning ability assessed by novel location test (NLT). $p = 0.8858$. All analyses were done using two-tailed, unpaired Student's *t* test, *sgCtrl*: N = 8 mice, *sgMap1b*: N = 7 mice. All data are presented as mean ± s.e.m. Source data are provided as a Source Data file.

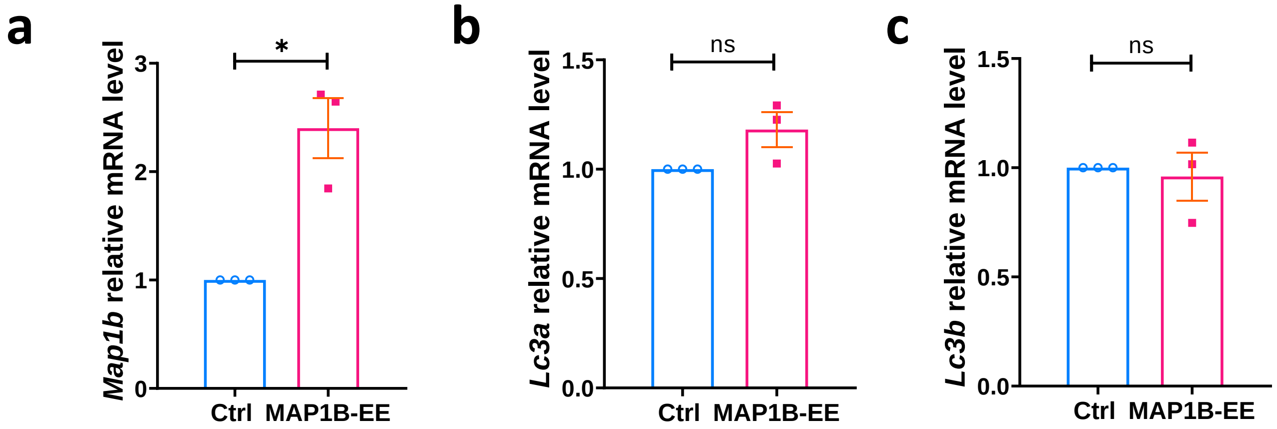

Supplementary Fig. 7 | MAP1B-EE does not have significant effect on *Lc3* mRNA levels in neurons. Quantitative qPCR analyses of *Map1b* (**a**), *Lc3a* (**b**) and *Lc3b* (**c**) mRNA levels in mouse hippocampal neurons with MAP1B-EE compared to control neurons. Two-tailed Student's *t* test with unequal variances, *Map1b*: $p = 0.0373$; *Lc3a*, $p = 0.151$; *Lc3b*, $p = 0.7489$. N = 3 biologically independent isolations. All error bars reflect mean ± s.e.m. Source data are provided as a Source Data file.

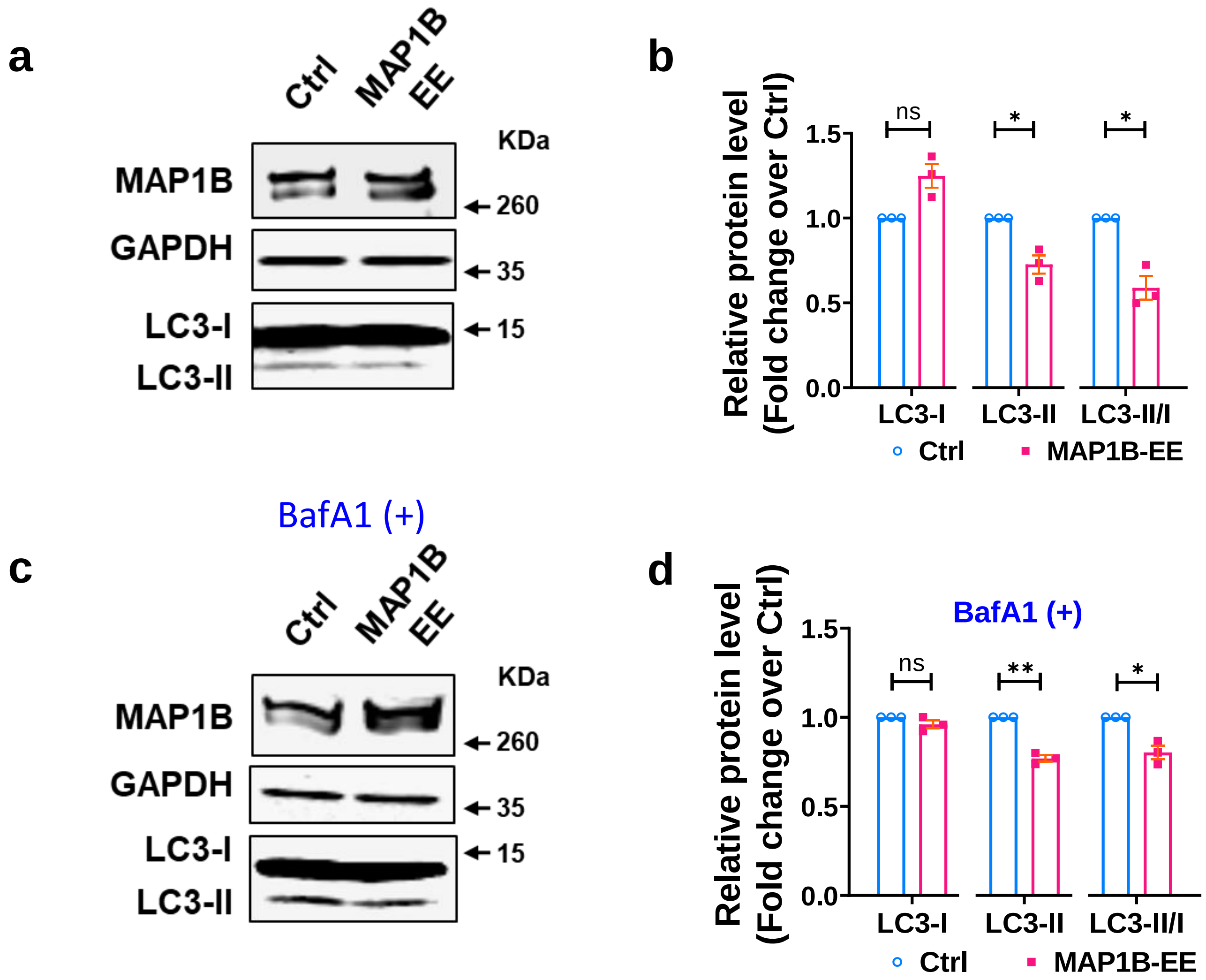

Supplementary Fig. 8 | MAP1B-EE leads to autophagy deficits in mouse primary cortical neurons. a, Sample Western blot analysis of cortical neurons with MAP1B-EE (LV-*sgMap1b infected*) compared to controls (*sgCtrl*). Neurons isolated from dCas9Activator mice infected with LV-*sgMAP1B* or LV-*sgCtrl* were harvest at DIV7. GAPDH was used as loading control. **b**, Quantitative analysis of LC3-I: p = 0.0698; LC3-II: p = 0.0366. LC3-II/I (LC3-II/LC3-I): p = 0.0274. **c,** Sample Western blot analysis of cortical neurons with MAP1B-EE and controls treated with BafA1 or vehicle at DIV7 for 6 hours before harvest. GAPDH was used as loading control. **d**, Quantitative analysis of proteins treated with BafA1 before harvest. LC3I: p = 0.2217; LC3-II: p = 0.0059. LC3-II/I: p = 0.0341. For quantification, protein amounts were normalized to GAPDH and subsequently normalized to control cells. Two-tailed, unpaired Student's t test with unequal variances was used. N = 3 biologically independent isolation of mouse neurons. All error bars reflect mean ± s.e.m. Source data are provided as a Source Data file.

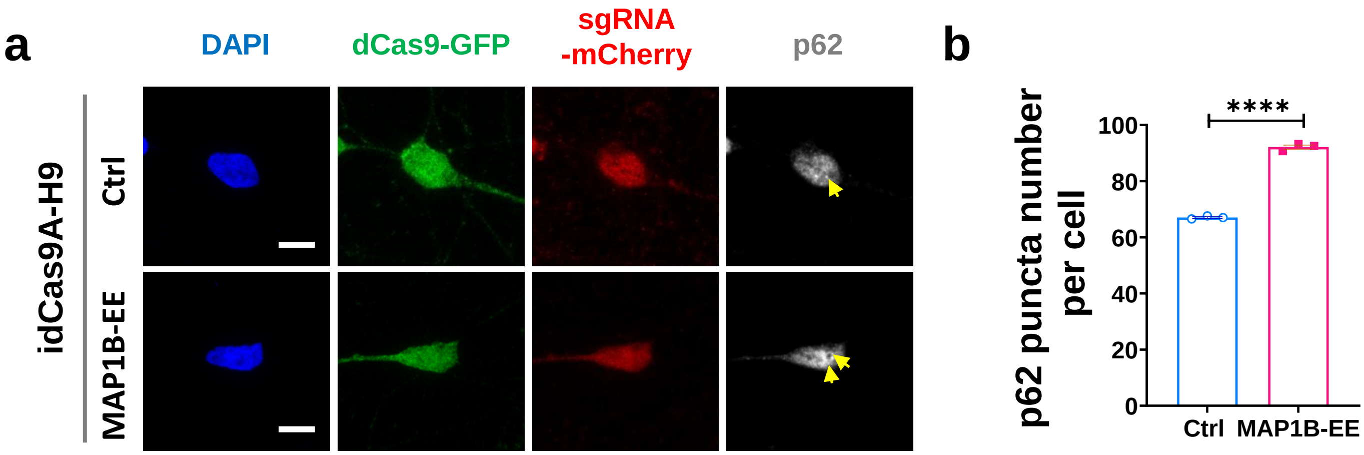

Supplementary Fig. 9 | Elevated MAP1B leads to p62 accumulation. a, Representative confocal images of p62 (white) puncta localized in idCas9A-H9 neurons with or without MAP1B-EE. Scale bars: 10 µm. **b,** Quantification of p62 puncta were done after 3D reconstruction. Two-tailed, unpaired Student's *t* test, $p < 0.0001$. n = 3 independent differentiation, N = 1. Data are presented as mean ± s.e.m. Arrow indicates p62 puncta. Source data are provided as a Source Data file.

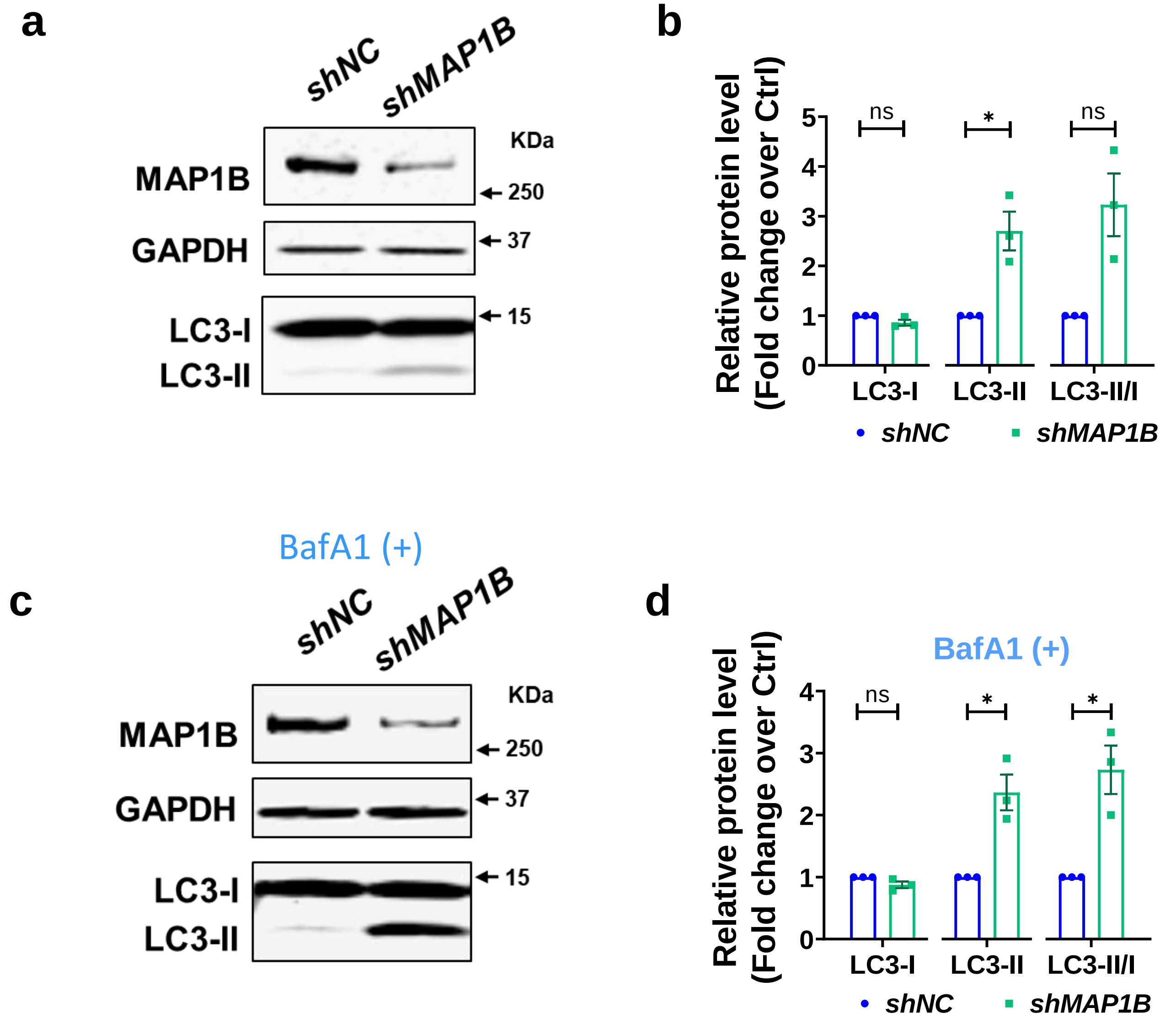

Supplementary Fig. 10 | MAP1B knockdown in 5q13.2trip ASD patient iPSC-derived neuron leads to increased LC3-II levels. a, Sample Western blot analysis of ASD neurons with MAP1B knockdown (*shMAP1B*) compared to control (*shNC).* **b,** Quantitative analysis of LC3I: $p = 0.1573$; LC3-II: $p = 0.0483$. LC3-II/LC3-I, $p = 0.0783$. **c**, Sample Western blot analysis of ASD neurons with or without MAP1B knockdown treated with BafA1. Neurons were treated with BafA1 for 6 hours before harvest. **d,** Quantitative analysis of LC3I: $p = 0.1448$; LC3-II: $p = 0.0419$. LC3-II/I: $p = 0.0471$. For quantification, protein amounts were normalized to GAPDH and subsequently normalized to control cells. Data were analyzed using two-tailed Student's *t* test with unequal variances. n = 3 independent differentiations, N = 1. All error bars reflect mean ± s.e.m. Source data are provided as a Source Data file.

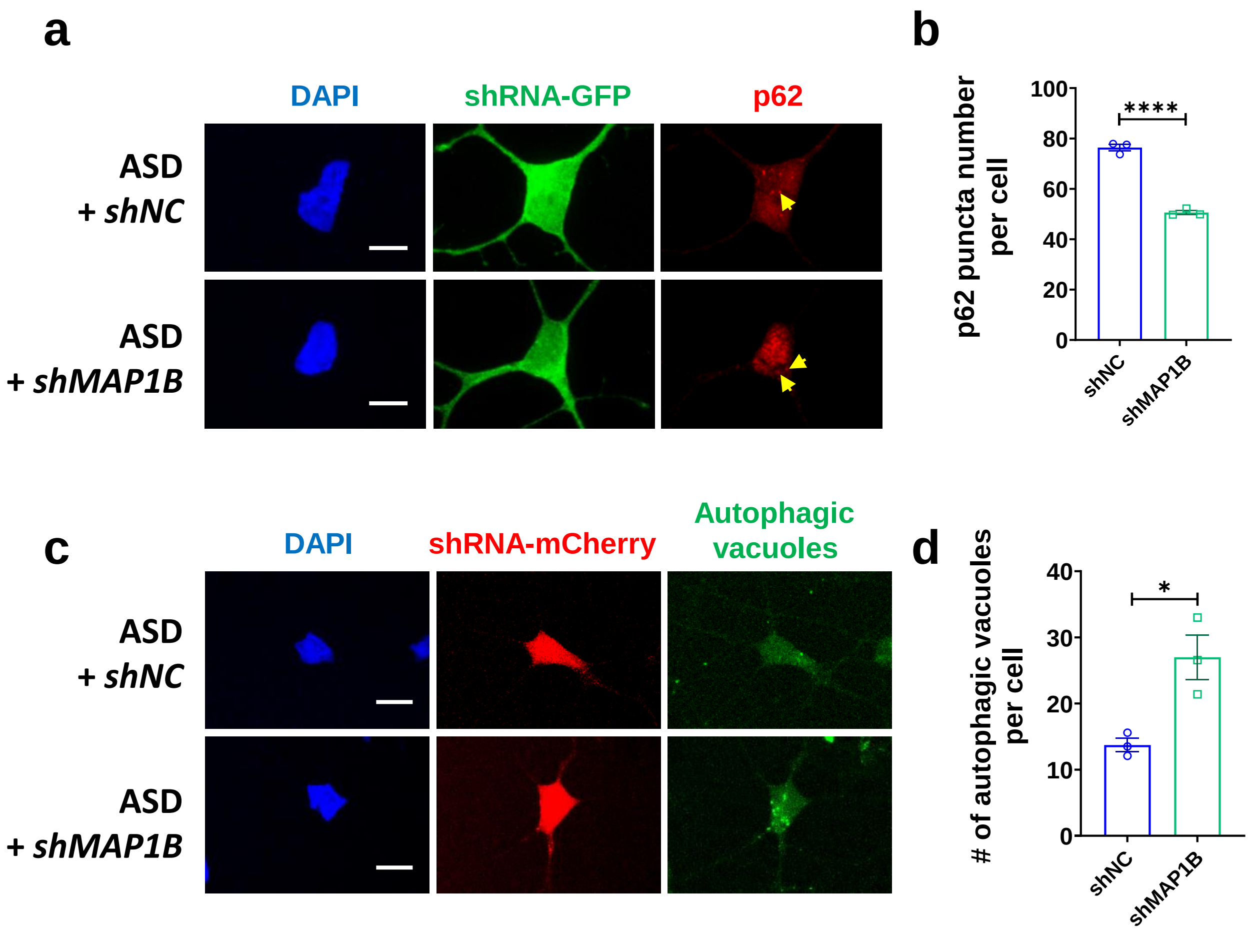

Supplementary Fig. 11 | MAP1B knockdown in 5q13.2trip ASD patient iPSC-derived neuron leads to reduced p62 accumulation and increased autophagy activity. a, Representative confocal images of p62 (red) puncta localized in ASD neurons with or without MAP1B knockdown. Scale bars: 10 µm. **b,** Quantifications of p62 puncta were done after 3D reconstruction. Two-tailed, unpaired Student's *t* test, $p < 0.0001$. n = 3 independent differentiation, N = 1. **c,** Representative confocal images of autophagic vacuoles (green puncta) localized in ASD neurons with or without MAP1B knockdown. Scale bars: 10 µm. **d,** Quantifications of autophagic vacuoles were done after 3D reconstruction. Two-tailed, unpaired Student's *t* test, $p = 0.0194$. n = 3 independent differentiation, N = 1. All data are presented as mean ± s.e.m. Arrow indicates p62 puncta. Source data are provided as a Source Data file.

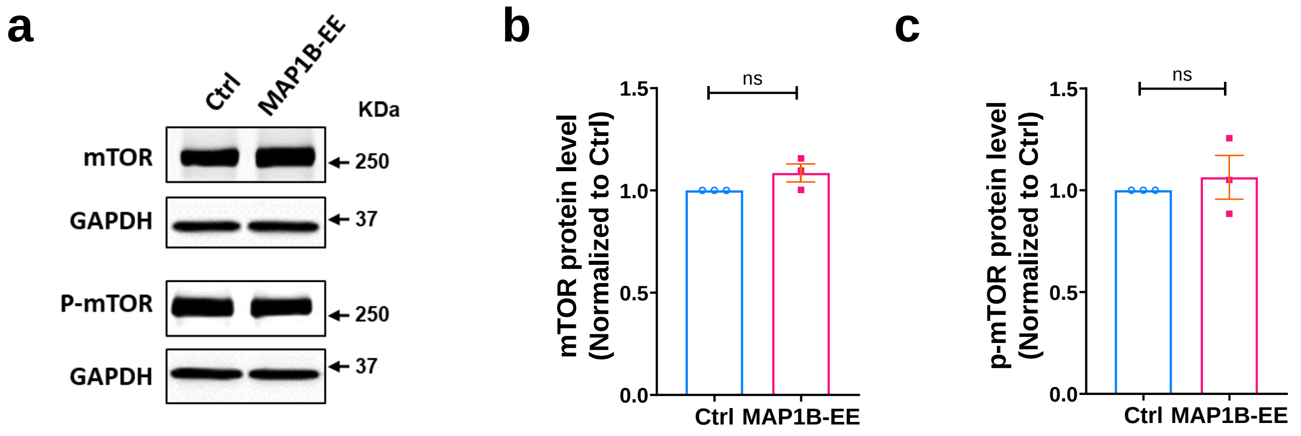

Supplementary Fig. 12 | MAP1B-EE neurons do not show significant changes in mTOR activation. a, Western blotting against mTOR and p-mTOR in dCas9Activator hippocampal neurons with or without MAP1B-EE. GAPDH was used as endogenous loading control. For quantification, protein amounts were normalized to GAPDH and subsequently compared to control cells. **b,c,** Quantification of mTOR, p = 0.1926 (**b**) and p-mTOR levels, p = 0.692 (**c**). N = 3 biologically independent isolations. Unpaired Student's t test with unequal variances was used. . All error bars reflect mean ± s.e.m. Source data are provided as a Source Data file.

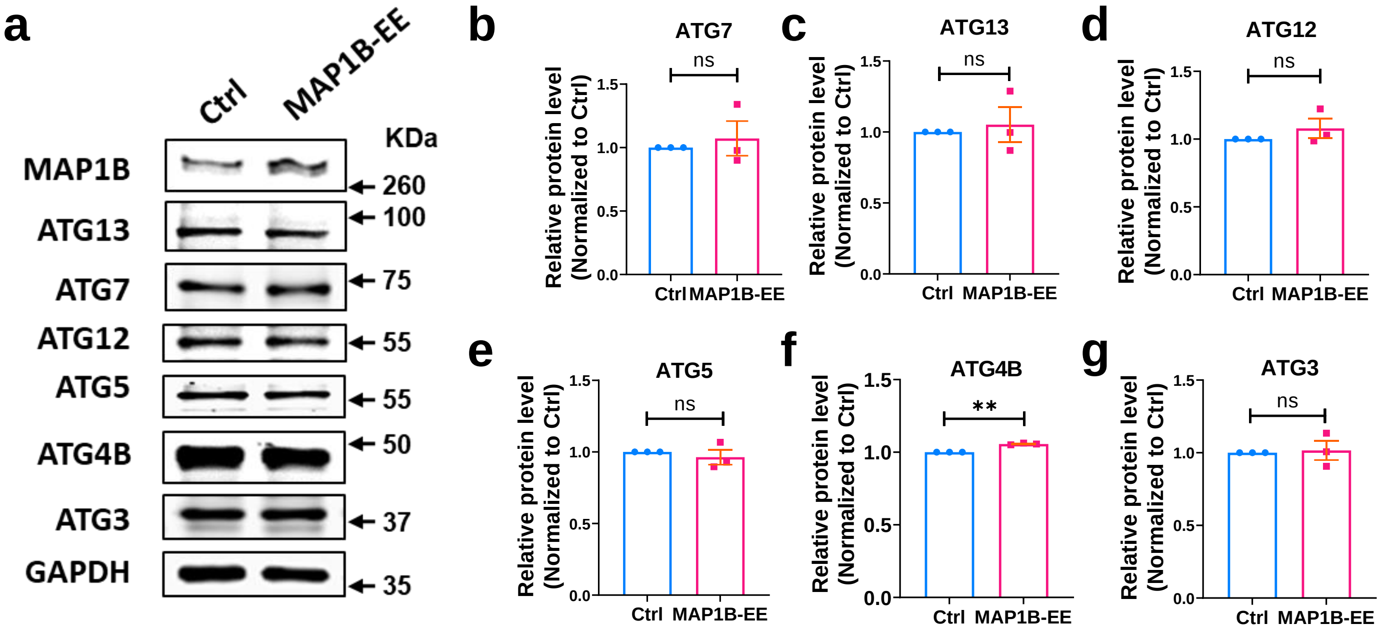

Supplementary Fig. 13 | MAP1B-EE does not have significant effect on the levels of proteins involved in LC3 lipidation. a, Representative Western blot images of dCas9Activator hippocampal neurons with MAP1B-EE (*sgMap1b*) or control (*sgCtrl*). GAPDH was used as endogenous loading control. For quantification in **b-g**, protein amounts were normalized to GAPDH and subsequently compared to control cells. **b,** Quantitative analysis of ATG13, p = 0.7169. **c,** ATG7, p = 0.6466. **d,** ATG12, p = 0.3879. **e,** ATG5, p = 0.5629. **f,** ATG4B, p = 0.0033, MAP1B-EE = 1.057 ± 0.003271. **g,** ATG3, p = 0.8287. N = 3 biologically independent isolations. The samples derive from the same experiment and the gels/blots were processed in parallel. Two-tailed, unpaired Student's t test with unequal variances was used. All error bars reflect mean ± s.e.m. Source data are provided as a Source Data file.

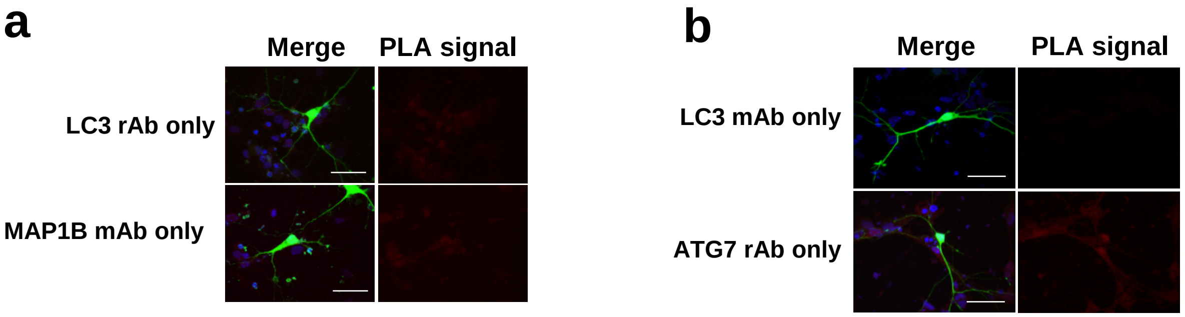

Supplementary Fig. 14 | Negative controls for PLA Experiments in Fig. 6. a, The neurons isolated from dCas9Activator mice (DIV 7) were incubated with either LC3 rabbit antibody or MAP1B mouse antibody alone. No PLA signal was detected. Scale bar, 50 µm. **b,** The neurons isolated from dCas9Activator mice (DIV 7) were incubated with either LC3 mouse antibody or ATG7 rabbit antibody alone. No PLA signal was detected. Scale bar, 50 µm.

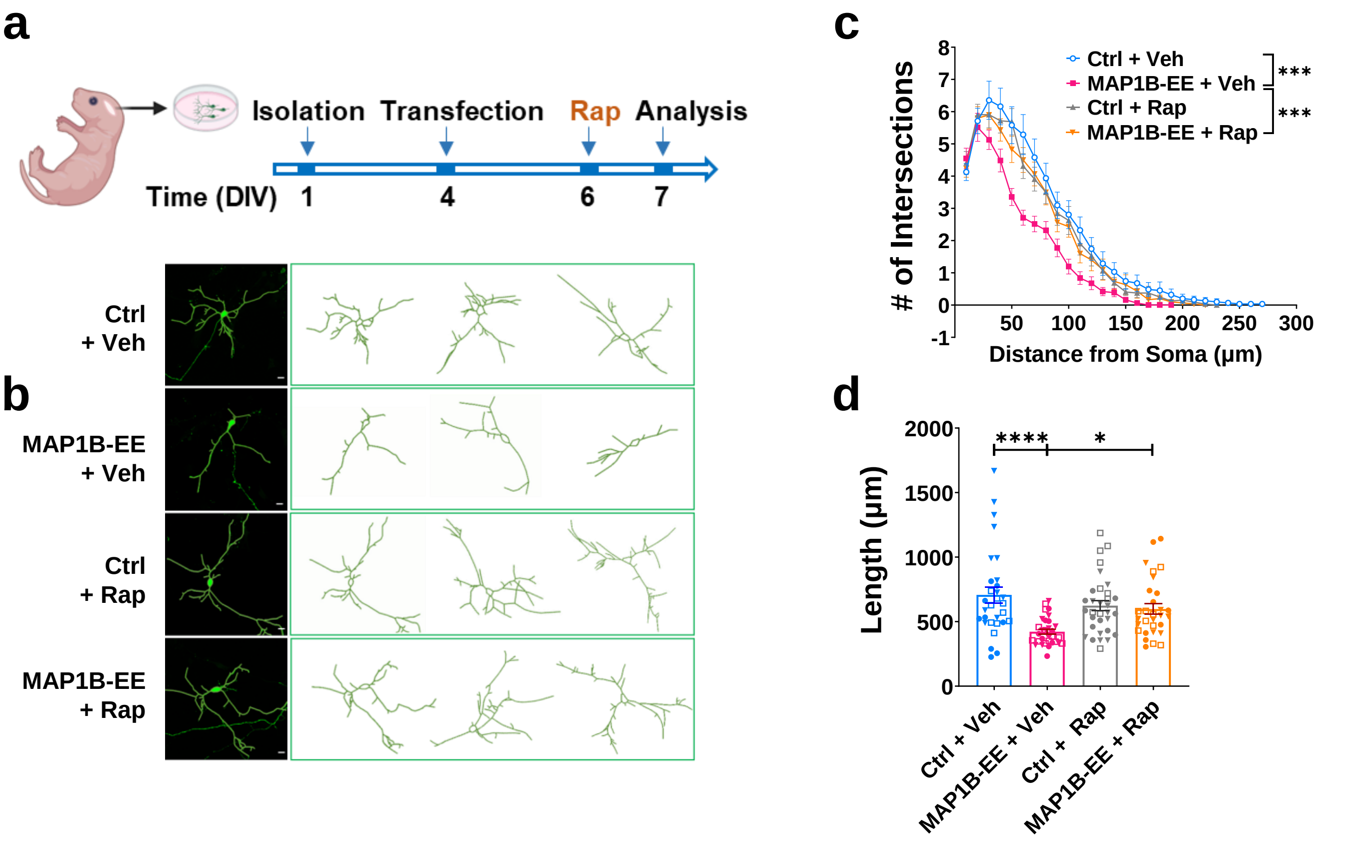

Supplementary Fig. 15 | Activation of autophagy pathway rescues MAP1B-EE-induced morphological deficits in mouse neurons. a, Experimental scheme for assessing the effect of autophagy activator rapamycin (Rap) on the dendritic maturation of MAP1B-EE mouse primary neurons. **b,** Representative confocal images (from N = 3 independently repeated experiments with similar results) and Neurolucida software-created traces of GFP+ neurons. Scale bar, 10 μm. **c,** Sholl analysis of dendritic complexity of MAP1B-EE or Ctrl neurons treated with DMSO (Vehicle, Veh) or rapamycin (Rap) (300 nM). MANOVA, Ctrl Veh vs. MAP1B-EE Veh: $F_{(1,59)} = 18.084$, $p < 0.001$; Ctrl Veh vs. Ctrl Rap: $F_{(1,60)} = 1.148$, $p = 0.288$; MAP1B-EE Veh vs. MAP1B-EE Rap: $F_{(1,59)} = 13.467$, $p < 0.001$. **d,** Quantification of total dendritic length of MAP1B-EE or Ctrl neurons treated with Veh or Rap. Two-way ANOVA with two-sided Bonferroni post hoc analysis for multiple comparisons, Ctrl Veh vs. MAP1B-EE Veh: $p < 0.0001$; MAP1B-EE Veh vs. MAP1B-EE Rap: $p = 0.0285$. For all data shown in (**c**) and (**d**), n = 30 neurons (Ctrl Veh); n = 32 neurons (Ctrl Rap); n = 31 neurons (MAP1B-EE Veh); n = 30 neurons (MAP1B-EE Rap). Data were from N = 3 independent neuronal isolations. Data are presented as mean ± s.e.m. Source data are provided as a Source Data file.

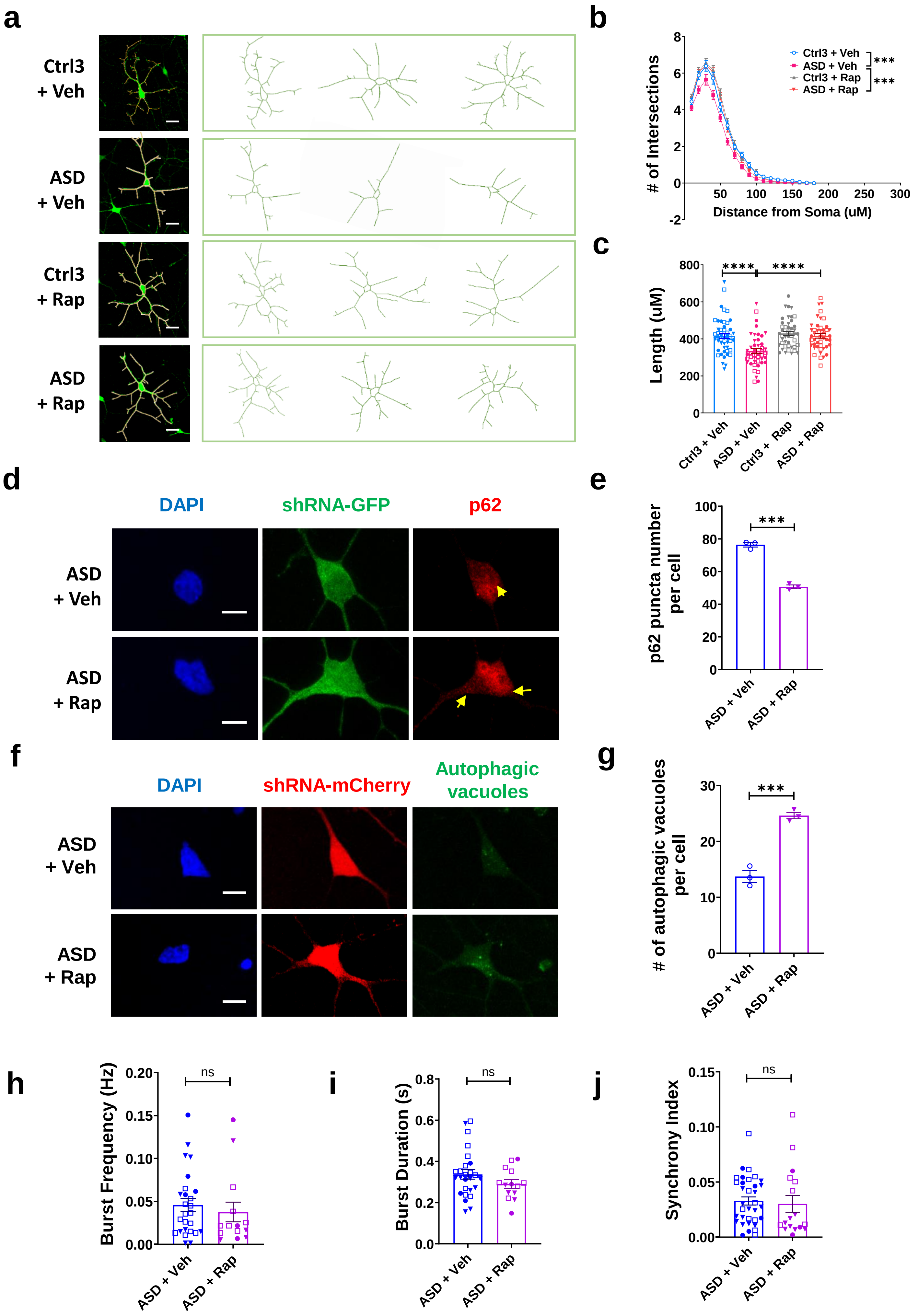
a
Ctrl3 + Veh
ASD + Veh
Ctrl3 + Rap
ASD + Rap
b
# of Intersections
Distance from Soma (uM)
Ctrl3 + Veh
ASD + Veh
Ctrl3 + Rap
ASD + Rap
***
***
c
Length (uM)
****
****
Ctrl3 + Veh
ASD + Veh
Ctrl3 + Rap
ASD + Rap
d
DAPI
shRNA-GFP
p62
ASD + Veh
ASD + Rap
e
p62 puncta number per cell
***
ASD + Veh
ASD + Rap
f
DAPI
shRNA-mCherry
Autophagic vacuoles
ASD + Veh
ASD + Rap
g
# of autophagic vacuoles per cell
***
ASD + Veh
ASD + Rap
h
Burst Frequency (Hz)
ns
ASD + Veh
ASD + Rap
i
Burst Duration (s)
ns
ASD + Veh
ASD + Rap
j
Synchrony Index
ns
ASD + Veh
ASD + Rap

Supplementary Fig. 16 | Rapamycin treatment rescues the morphological and autophagy deficits in 5q13.2trip ASD patient neurons. a, Representative confocal images (from 3 independently repeated experiments with similar results) and Neurolucida software-created traces of GFP+ neurons. Scale bar, 20 µm. **b,** Sholl analysis of dendritic complexity of Ctrl3 neurons (WC5907) or ASD neurons treated with DMSO (Vehicle, Veh) or rapamycin (Rap) (300 nM). MANOVA, Ctrl3 Veh vs. ASD Veh: $F_{(1,92)} = 19.327$, $p < 0.001$; Ctrl3 Veh vs. Ctrl3 Rap: $F_{(1,92)} = 0.962$, $p = 0.329$; ASD Veh vs. ASD Rap: $F_{(1,88)} = 20.073$, $p < 0.001$. **c,** Quantification of total dendritic length of ASD or Ctrl3 neurons treated with Veh or Rap. For all data shown in (**b**) and (**c**), n = 49 neurons (Ctrl3 Veh); n = 45 neurons (Ctrl3 Rap); n = 45 neurons (ASD Veh); n = 45 neurons (ASD Rap). Data were from 3 independent neuronal differentiation, N = 1. Two-way ANOVA with two-sided Bonferroni post hoc analysis for multiple comparisons, $p < 0.0001$. **d,** Representative confocal images of p62 (red) puncta localized in ASD neurons with or without rapamycin treatment. Scale bars: 10 µm. **e,** Quantifications of p62 puncta were done after 3D reconstruction. Two-tailed, unpaired Student's *t* test, $p = 0.0001$. n = 3 independent differentiation, N = 1. **f,** Representative confocal images of autophagic vacuoles (green puncta) localized in ASD neurons with or without rapamycin treatment. Scale bars: 10 µm. **g,** Quantifications of autophagic vacuoles were done after 3D reconstruction. Two-tailed, unpaired Student's *t* test, $p = 0.0008$. n = 3 independent differentiation, N = 1. **h,i,** Quantifications of burst frequency **(h)**, $p = 0.5391$ and burst duration **(i)**, $p = 0.1821$. Two-tailed, unpaired Student's *t* test. Only the wells with bursting activity were analyzed for burst-related parameters, ASD Veh: n = 26 individual wells, ASD Rap: n = 14 individual wells from 3 independent neuronal differentiations, N = 1. **j,** Quantifications of synchrony index, $p = 0.723$. Two-tailed, unpaired Student's *t* test. ASD Veh: n = 34 individual wells, ASD Rap: n = 17 individual wells from 3 individual neuronal differentiations, N = 1. All data are presented as mean ± s.e.m. Source data are provided as a Source Data file.

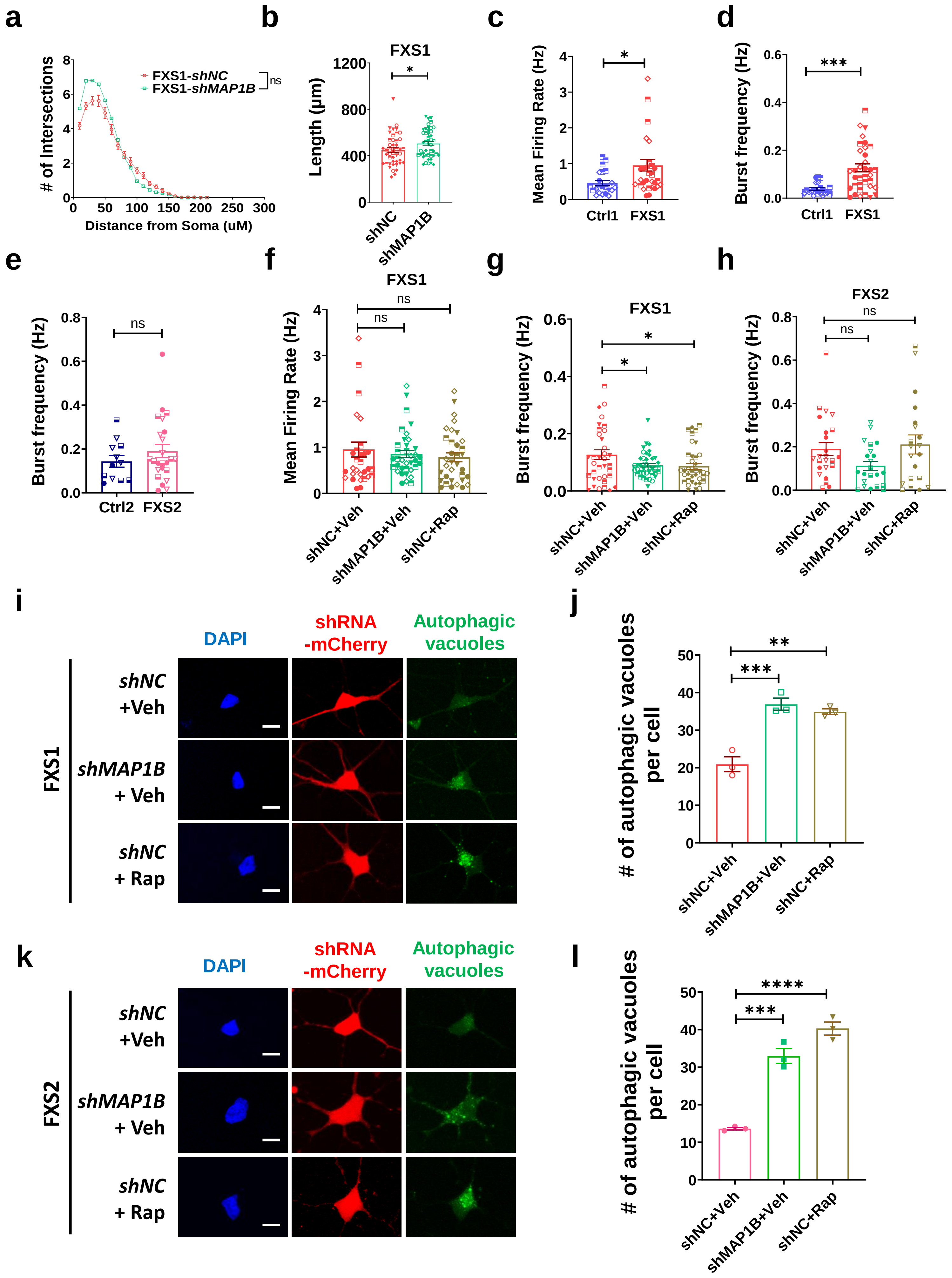

a
# of Intersections
Distance from Soma (uM)
FXS1-shNC
FXS1-shMAP1B
ns
b
FXS1
Length (µm)
*
shNC
shMAP1B
c
Mean Firing Rate (Hz)
*
Ctrl1
FXS1
d
Burst frequency (Hz)
***
Ctrl1
FXS1
e
Burst frequency (Hz)
ns
Ctrl2
FXS2
f
FXS1
Mean Firing Rate (Hz)
ns
ns
shNC+Veh
shMAP1B+Veh
shNC+Rap
g
FXS1
Burst frequency (Hz)
*
*
shNC+Veh
shMAP1B+Veh
shNC+Rap
h
FXS2
Burst frequency (Hz)
ns
ns
shNC+Veh
shMAP1B+Veh
shNC+Rap
i
DAPI
shRNA -mCherry
Autophagic vacuoles
FXS1
shNC +Veh
shMAP1B + Veh
shNC + Rap
j
# of autophagic vacuoles per cell
**
***
shNC+Veh
shMAP1B+Veh
shNC+Rap
k
DAPI
shRNA -mCherry
Autophagic vacuoles
FXS2
shNC +Veh
shMAP1B + Veh
shNC + Rap
l
# of autophagic vacuoles per cell
****
***
shNC+Veh
shMAP1B+Veh
shNC+Rap

Supplementary Fig. 17 | Effect of Rapamycin treatment on FMRP deficient human neurons. a, Sholl analysis of dendritic complexity of FXS1 neurons with (*shMAP1B*) or without (*shNC*) MAP1B knockdown. MANOVA, $F_{(1,88)} = 1.948$, $p = 0.166$. n = 45 neurons from 4 independent neuronal differentiations, N = 1. **b,** Quantification of total dendritic length. Two-tailed, unpaired Student's *t* test, $p = 0.0374$, n = 45 neurons from 4 independent neuronal differentiations, N = 1. **c,** Quantifications of neuronal mean firing rate in Ctrl1 and FXS1 neurons. Two-tailed, unpaired Student's *t* test, $p = 0.0195$. Ctrl1: n = 22 individual wells, FXS1: n = 33 individual wells from 4 individual neuronal differentiations, N = 1. **d,** Quantifications of burst frequency in Ctrl1 and FXS1 neurons. Two-tailed, unpaired Student's *t* test, $p = 0.0002$. Only the wells with bursting activity were analyzed for burst-related parameters, Ctrl1: n = 21 individual wells; FXS1: n = 37 individual wells from 4 independent neuronal differentiations, N = 1. **e,** Quantifications of burst frequency in Ctrl2 and FXS2 neurons. Two-tailed, unpaired Student's *t* test, $p = 0.3236$. Only the wells with bursting activity were analyzed for burst-related parameters, Ctrl2: n = 12 individual wells; FXS2: n = 24 individual wells from 3 independent neuronal differentiations, N = 1. **f,** Quantifications of neuronal mean firing rate in FXS1 neurons with MAP1B knockdown or rapamycin treatment. One-way ANOVA with Dunnett post hoc tests, *shNC*+Veh vs. *shMAP1B*+Veh: $p = 0.7666$; *shNC*+Veh vs. *shNC*+Rap: $p = 0.4874$. *shNC* Veh: n = 33 individual wells, *shMAP1B* Veh: n = 37 individual wells, *shNC* Rap: n = 34 wells from 4 independent neuronal differentiations, N = 1. **g,** Quantifications of burst frequency in FXS1 neurons with MAP1B knockdown or rapamycin treatment. One-way ANOVA with Dunnett post hoc tests, *shNC*+Veh vs. *shMAP1B*+Veh: $p = 0.0476$; *shNC*+Veh vs. *shNC*+Rap: $p = 0.0316$. Only the wells with bursting activity were analyzed for burst-related parameters, *shNC* Veh: n = 37 individual wells, *shMAP1B* Veh: n = 42 individual wells, *shNC* Rap: n = 38 wells from 4 independent neuronal differentiations, N = 1 **h,** Quantifications of burst frequency in Ctrl2 and FXS2 neurons. One-way ANOVA with Dunnett post hoc tests, *shNC*+Veh vs. *shMAP1B*+Veh: $p = 0.1538$; *shNC*+Veh vs. *shNC*+Rap: $p = 0.8583$. Only the wells with bursting activity were analyzed for burst-related parameters, *shNC* Veh: n = 24 individual wells, *shMAP1B* Veh: n = 22 individual wells, *shNC* Rap: n = 20 wells from 3 individual neuronal differentiations, N = 1. **i,** Representative confocal images of autophagic vacuoles (green puncta) localized in FXS1 neurons with MAP1B knockdown or rapamycin treatment. Scale bars: 10 μm. **j,** Quantifications of autophagic vacuoles were done after 3D reconstruction. One-way ANOVA with Dunnett post hoc tests, *shNC*+Veh vs. *shMAP1B*+Veh: $p = 0.0006$; *shNC*+Veh vs. *shNC*+Rap: $p = 0.0012$. n = 3 independent differentiation, N = 1. **k,** Representative confocal images of autophagic vacuoles (green puncta) localized in FXS2 neurons with MAP1B knockdown or rapamycin treatment. Scale bars: 10 μm. **l,** Quantifications of autophagic vacuoles were done after 3D reconstruction. One-way ANOVA with Dunnett post hoc tests, *shNC*+Veh vs. *shMAP1B*+Veh: $p = 0.0002$; *shNC*+Veh vs. *shNC*+Rap: $p < 0.0001$. n = 3 independent differentiation of each line, N = 1. All data are presented as mean ± s.e.m. Source data are provided as a Source Data file.

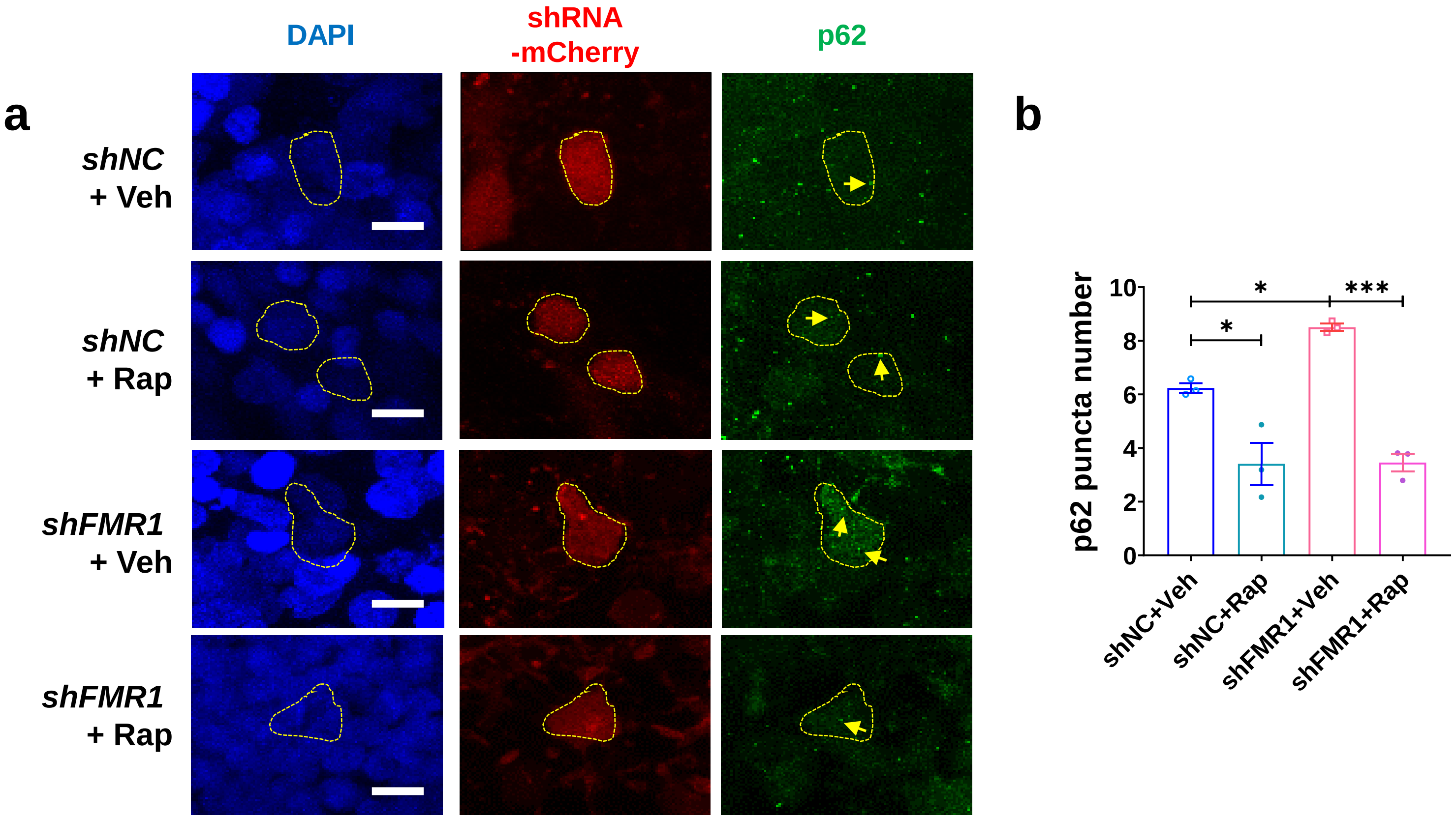

Supplementary Fig. 18 | FMRP deficiency leads to p62 accumulation in neurons in human mid-fetal cortical tissue. a, Representative confocal images of neurons in the cortex expressing shRNA-mCherry (red), p62 (green) in lentivirus-infected ex vivo human cortical slices. Scale bars: 10 µm. **b,** Quantifications of p62 puncta were done after 3D reconstruction in mCherry+ neurons in human cortical slices. Two-way ANOVA with two-sided Bonferroni post hoc analysis for multiple comparisons: *shNC*+Veh vs. *shNC*+Rap: $p = 0.0114$; *shNC*+Veh vs. *shFMR1*+Veh: $p = 0.0402$; *shFMR1*+Veh vs. *shFMR1*+Rap: $p = 0.0002$. N = 3 individual cortices. All data are presented as mean ± s.e.m. Source data are provided as a Source Data file.

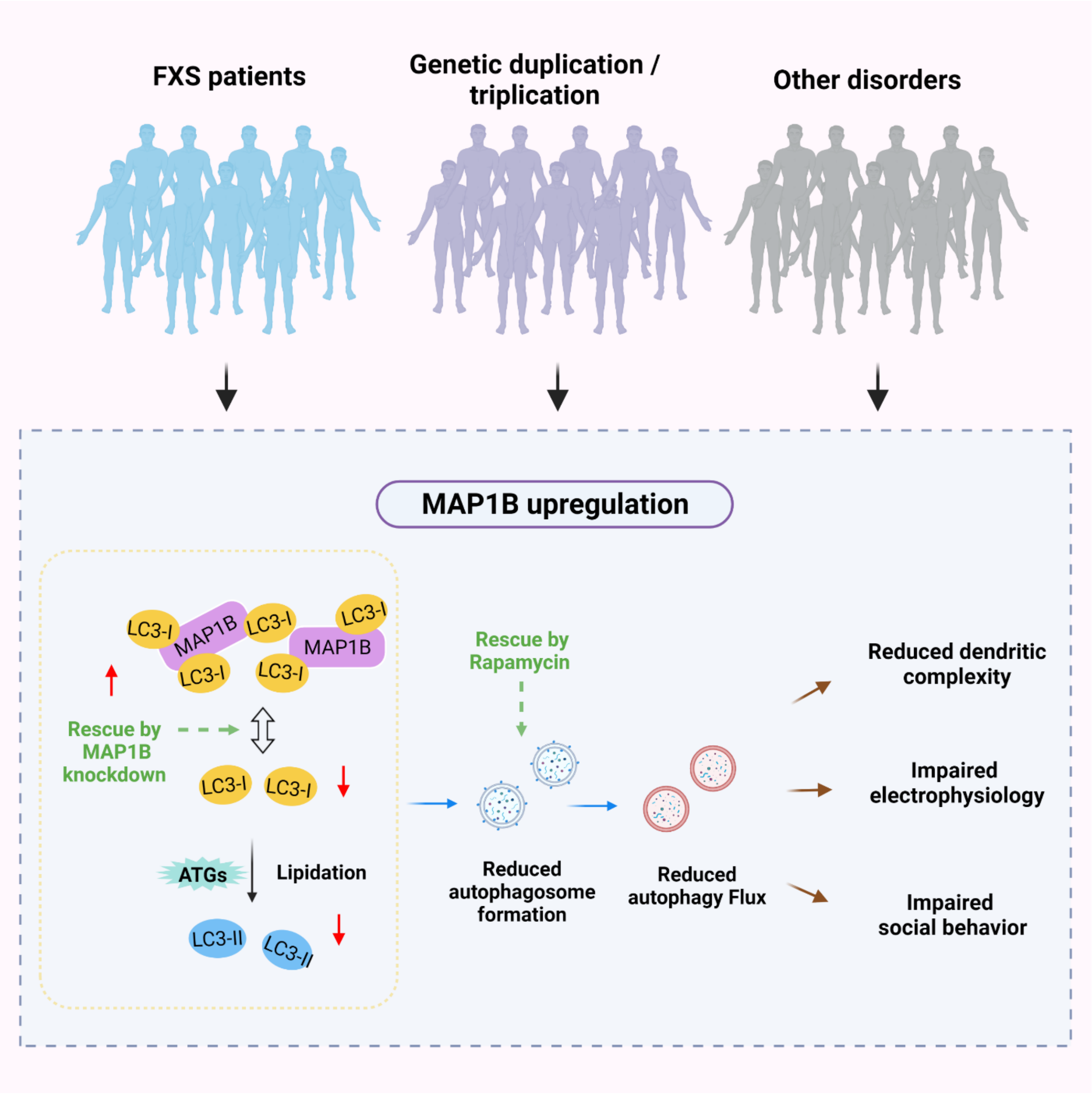

Supplementary Fig. 19 | A schematic model illustrating how MAP1B-EE impairs neuronal development and social behaviors

In healthy neurons, there is an equilibrium between MAP1B-bonded LC3-I and freely available LC3-I. The freely available LC3-I can interact with ATG proteins that add phospholipids to LC3-I to form LC3-II (lipidation) and initiate the autophagosome formation. In neurons with MAP1B-EE, high levels of MAP1B bind and sequester cellular LC3-I and reduce the amount of LC3-I that can interact with ATGs leading to reduced autophagosome formation and impaired autophagy. Rapamycin increases LC3-II levels through inhibiting mTOR signaling. In FXS and ASD neurons with elevated MAP1B levels, both genetic reduction of MAP1B and treatment with rapamycin can rescue autophagy therefore rescue morphological and electrophysiological deficits.
