# Supplementary material for: Elevated levels of FMRP-target MAP1B impair human and mouse neuronal development and mouse social behaviors via autophagy pathway
Source: Nat Commun. 2023 Jun 26;14:3801. doi: 10.1038/s41467-023-39337-0 (PMC10293283; doi:10.1038/s41467-023-39337-0)
Supplement: Supplementary file 3 — Description of Additional Supplementary Files [file 41467_2023_39337_MOESM3_ESM.pdf]

Description of Additional Supplementary Files

File Name: Supplementary Data 1
Description: Information for cell lines and animal models

File Name: Supplementary Data 2
Description: Summary of 5q13.2 CNV

File Name: Supplementary Data 3
Description: Plasmids, primers, antibodies used in this paper
