# Supplementary material for: Elevated levels of FMRP-target MAP1B impair human and mouse neuronal development and mouse social behaviors via autophagy pathway
Source: Nat Commun. 2023 Jun 26;14:3801. doi: 10.1038/s41467-023-39337-0 (PMC10293283; doi:10.1038/s41467-023-39337-0)
Supplement: Supplementary file 7 — Reporting Summary [file 41467_2023_39337_MOESM7_ESM.pdf]

nature portfolio

Corresponding author(s): Xinyu Zhao

Last updated by author(s): May 5, 2023

Reporting Summary

Nature Portfolio wishes to improve the reproducibility of the work that we publish. This form provides structure for consistency and transparency in reporting. For further information on Nature Portfolio policies, see our Editorial Policies and the Editorial Policy Checklist.

Statistics

For all statistical analyses, confirm that the following items are present in the figure legend, table legend, main text, or Methods section.

n/a	Confirmed	
☐	☒	The exact sample size (*n*) for each experimental group/condition, given as a discrete number and unit of measurement
☐	☒	A statement on whether measurements were taken from distinct samples or whether the same sample was measured repeatedly
☐	☒	The statistical test(s) used AND whether they are one- or two-sided<br>*Only common tests should be described solely by name; describe more complex techniques in the Methods section.*
☐	☒	A description of all covariates tested
☐	☒	A description of any assumptions or corrections, such as tests of normality and adjustment for multiple comparisons
☐	☒	A full description of the statistical parameters including central tendency (e.g. means) or other basic estimates (e.g. regression coefficient) AND variation (e.g. standard deviation) or associated estimates of uncertainty (e.g. confidence intervals)
☐	☒	For null hypothesis testing, the test statistic (e.g. *F*, *t*, *r*) with confidence intervals, effect sizes, degrees of freedom and *P* value noted<br>*Give P values as exact values whenever suitable.*
☒	☐	For Bayesian analysis, information on the choice of priors and Markov chain Monte Carlo settings
☒	☐	For hierarchical and complex designs, identification of the appropriate level for tests and full reporting of outcomes
☒	☐	Estimates of effect sizes (e.g. Cohen's *d*, Pearson's *r*), indicating how they were calculated

Our web collection on statistics for biologists contains articles on many of the points above.

Software and code

Policy information about availability of computer code

Data collection	NIS-Elements 5.41.01 for confocal imaging (Nikon); Image studio 5.2 for scanning western blot (LICOR); pClamp 10.4 for whole-cell patch-clamp recordings (Molecular Devices); Quantity One 4.6.6 for imaging regular genotyping gels (BioRad); AxIS Navigator 3.5 for MEA recording (Axion Biosystems); Fusion Accuscan program for open field tests (Omnitech Electronics).
Data analysis	Neurolucida 10 was used for 2D neuronal tracing, Neurolucida 2021 was used for 3D neuronal tracing, FIJI (Image J) for imaging analysis (RRID: SCR_003070), 3D reconstruction was conducted using FIJI (Image J) software with Clear Volume plug-in, ANY-maze 7.1 (Stoelting) was used for behavior test data analysis, SPSS version 28 and GraphPad Prism 9 were used for statistical analysis.

For manuscripts utilizing custom algorithms or software that are central to the research but not yet described in published literature, software must be made available to editors and reviewers. We strongly encourage code deposition in a community repository (e.g. GitHub). See the Nature Portfolio guidelines for submitting code & software for further information.

Data

Policy information about availability of data

All manuscripts must include a data availability statement. This statement should provide the following information, where applicable:

- Accession codes, unique identifiers, or web links for publicly available datasets
- A description of any restrictions on data availability
- For clinical datasets or third party data, please ensure that the statement adheres to our policy

The data that support the findings of this study are available from the corresponding author upon reasonable request

Research involving human participants, their data, or biological material

Policy information about studies with human participants or human data. See also policy information about sex, gender (identity/presentation), and sexual orientation and race, ethnicity and racism.

Reporting on sex and gender	*Use the terms sex (biological attribute) and gender (shaped by social and cultural circumstances) carefully in order to avoid confusing both terms. Indicate if findings apply to only one sex or gender; describe whether sex and gender were considered in study design; whether sex and/or gender was determined based on self-reporting or assigned and methods used. Provide in the source data disaggregated sex and gender data, where this information has been collected, and if consent has been obtained for sharing of individual-level data; provide overall numbers in this Reporting Summary. Please state if this information has not been collected. Report sex- and gender-based analyses where performed, justify reasons for lack of sex- and gender-based analysis.*
Reporting on race, ethnicity, or other socially relevant groupings	*Please specify the socially constructed or socially relevant categorization variable(s) used in your manuscript and explain why they were used. Please note that such variables should not be used as proxies for other socially constructed/relevant variables (for example, race/ethnicity should not be used as a proxy for socioeconomic status). Provide clear definitions of the relevant terms used, how they were provided (by the participants/respondents, the researchers, or third parties), and the method(s) used to classify people into the different categories (e.g. self-report, census or administrative data, social media data, etc.) Please provide details about how you controlled for confounding variables in your analyses.*
Population characteristics	*Describe the covariate-relevant population characteristics of the human research participants (e.g. age, genotypic information, past and current diagnosis and treatment categories). If you filled out the behavioural & social sciences study design questions and have nothing to add here, write "See above."*
Recruitment	*Describe how participants were recruited. Outline any potential self-selection bias or other biases that may be present and how these are likely to impact results.*
Ethics oversight	*Identify the organization(s) that approved the study protocol.*

Note that full information on the approval of the study protocol must also be provided in the manuscript.

Field-specific reporting

Please select the one below that is the best fit for your research. If you are not sure, read the appropriate sections before making your selection.

☒ Life sciences ☐ Behavioural & social sciences ☐ Ecological, evolutionary & environmental sciences

For a reference copy of the document with all sections, see nature.com/documents/nr-reporting-summary-flat.pdf

Life sciences study design

All studies must disclose on these points even when the disclosure is negative.

Sample size	The sample size was determined based on power analyses (StateMate), our publications (ref 31,33,39,65,82,96), and literature (ref 97-101). Please see the Reference section for detailed citations.
Data exclusions	The data exclusion criteria was pre-established before the data was acquired.<br>Wells from MEA recording experiments were excluded from analysis based on publications (ref 93, 94)<br>1. Wells with < 25% active electrodes or MFR < 0.1 Hz were not included in data analysis.<br>2. Wells that displayed insufficient quality, for example, cell clumping, were discarded<br>3. Wells were identified as outliers by the ROUT method with Q=1%<br>Cells were excluded in sholl analysis if they appeared as clear outliers and identified as outliers by the ROUT method with Q=1%<br>No data were excluded in the other data analyses.
Replication	At least two experimental replicates were performed for each biological sample. Replication of all experiments was successful.
Randomization	All samples of the same genotype were randomly assigned for the control and experimental groups and all cells analyzed were randomly

Randomization	selected from in vivo and in vitro samples.
Blinding	Data collection and quantifications were performed by experimenters who were blind to the identity of the samples.

Reporting for specific materials, systems and methods

We require information from authors about some types of materials, experimental systems and methods used in many studies. Here, indicate whether each material, system or method listed is relevant to your study. If you are not sure if a list item applies to your research, read the appropriate section before selecting a response.

Materials & experimental systems

n/a	Involved in the study
☐	☒ Antibodies
☐	☒ Eukaryotic cell lines
☒	☐ Palaeontology and archaeology
☐	☒ Animals and other organisms
☒	☐ Clinical data
☒	☐ Dual use research of concern
☒	☐ Plants

Methods

n/a	Involved in the study
☒	☐ ChIP-seq
☒	☐ Flow cytometry
☒	☐ MRI-based neuroimaging

Antibodies

Antibodies used

Antibody Distributer Cat# Dilution
MAP1B Santa Cruz sc-365668 (Clone: H8) 1:1000 (WB), 1:500 (PLA)
LC3 Novus Biologicals NB100-2331 1:1000 (WB), 1:200 (PLA)
LC3 Santa Cruz sc-271625 (Clone: G2) 1:1000 (WB), 1:25 (PLA)
ATG7 ABclonal A19604 (Clone: ARC0083) 1:1000 (WB), 1:50 (PLA)
p62 MBL International PM045 (Clone: 5F2) 1:1000
mTOR Cell Signaling Technology 2983T (Clone: 7C10) 1:1000
p-mTOR Cell Signaling Technology 5536T (Clone: D9C2) 1:1000
ATG13 ABclonal A0690 1:1000
ATG12 ABclonal A19610 (Clone: ARC0089) 1:1000
AGT5 ABclonal A19677 (Clone: ARC0156) 1:1000
ATG4B ABclonal A5059 (Clone: ARC1264) 1:1000
ATG3 ABclonal A19594 (Clone: ARC0073) 1:1000
GAPDH Thermo Fisher Scientific TAB1001 1:2000
GAPDH Thermo Fisher Scientific MA5-15738 (Clone: GA1R) 1:2000
GFP Abcam ab290 1:3000
GFP Thermo Fisher Scientific A-11120 (Clone: 3E6) 4ug (IP)
GFP Thermo Fisher Scientific A10262 1:2000
mCherry Thermo Fisher Scientific M11217 (Clone: 16D7) 1:2000
OCT4 (POU5F1) Santa Cruz sc-5279 (Clone: C-10) 1:1000
SOX2 R&D System MAB2018 (Clone: # 245610) 1:1000
TRA1-81 (PODXL) Millipore MAB4381 (Clone: TRA-1-81) 1:1000
PAX6 Biolegend 901301 1:500
TUBB3 Promega G712A (Clone: 5G8) 1:2000
vGlut1 (SLC17A7) SynapticSystems 135-303 1:2000

Validation

Antibodies were validated by the manufacturers or the publication using WB.

Eukaryotic cell lines

Policy information about cell lines and Sex and Gender in Research

Cell line source(s)	HEK293T cells (ATCC, CRL-3216), Neuro2A cells (ATCC), H9 human embryonic stem cells (WA09 from WiCell), H1 human embryonic stem cells (WA01, from WiCell), H13 human embryonic stem cells (WA13 from WiCell), GM1 human induced pluripotent stem cells (created by Waisman Center Core, published in Li et al 2020), WC5907 human induced pluripotent stem cells (WiCell), WC6007 human induced pluripotent stem cells (WiCell), FX11-7 FXS patient induced pluripotent stem cells (WiCell), FX13-2 FXS patient induced pluripotent stem cells (WiCell), doxycycline inducible dCas9-Activator H9 human embryonic stem cell line (idCas9A-H9, created in this study), fibroblasts from 5q13.2trip ASD patient and his mother (created in this study, Induced pluripotent stem cell derived from 5q13.2trip ASD patient fibroblast (created in this study).
Authentication	HEK293T cells and Neuro2A cells have been authenticated by ATCC. H9, H1, H13, WC5907, WC6007, GM1, FX11-7, FX13-2, lines are authenticated by WiCell and published. Authentication of idCas9A-H9 and ASD patient iPSC lines have been done in this study (see supplementary figures)
Mycoplasma contamination	The cell lines used in this study were tested negative for mycoplasma contamination.

Commonly misidentified lines (See ICLAC register)	No misidentified line was used in this study.

Animals and other research organisms

Policy information about studies involving animals; ARRIVE guidelines recommended for reporting animal research, and Sex and Gender in Research

Laboratory animals	C57BL/6 and dCas9Activator mice, both male and female at different ages according to experimental design (P0 up to 4 months old), were housed in IACUC approved conditions. Rhesus macaque were PCD91, 101, 104; human midfetal brain tissue were at PCD 115, 115, 137.
Wild animals	No wild animals were used in this study
Reporting on sex	Both male and female neonatal mice were used in this study; only male young adult mice were used for behavioral tests; the sex of human fetal cortices were from postmortem female individuals. The sex of Rhesus macaque fetal cortices were not identified by Wisconsin Primate Center
Field-collected samples	No field-collected samples were used in this study
Ethics oversight	We performed all procedures involving live mice in accordance with the NIH Guide for the Care and Use of Laboratory Animals and the protocols approved by the University of Wisconsin-Madison Animal Care and Use Committee (IACUC).<br>Human (Homo sapiens) fetal tissue was collected by the Birth Defects Research Laboratory at the University of Washington in compliance with all Federal, State and Institutional regulations. Donors are recruited at clinical sites in the Puget Sound area under an approved human subjects research protocol at the University of Washington. After a patient has consented in writing to pregnancy termination with a clinical provider, she is then consented for research participation by a second staff member trained in human subjects research and HIPAA regulations. The research consent form is signed by the participant and the second staff member. Research participation does not affect the method of termination, nor are enticements of any type provided to the participant, clinic staff, or research team members. We used de-identified postmortem human brain specimens. This study was performed in accordance with ethical and legal guidelines of the University of Wisconsin-Madison Institutional Review Board. Appropriate informed consent was obtained and all available non-identifying information was recorded for each specimen. Tissue was handled in accordance with ethical guidelines and regulations for the research use of human brain tissue set forth by the NIH (http://bioethics.od.nih.gov/humantissue.html) and the WMA Declaration of Helsinki (http://www.wma.net/en/30publications/10policies/b3/index.html).<br>All experiments using non-human primates Rhesus macaque (Macaca mulatta) (91, 101 and 104 PCD) were carried out in accordance with a protocol approved by University of Wisconsin's Institutional Animal Care and Use Committee and NIH guidelines. All clinical histories, tissue specimens, and histological sections were evaluated to assess for signs of disease, injury, and gross anatomical and histological alterations. No obvious signs of neuropathological alterations were observed in any of the macaque specimens analyzed in this study.

Note that full information on the approval of the study protocol must also be provided in the manuscript.
